# Experiment-guided molecular simulations define a heterogeneous structural ensemble for the *PTPN11* tandem SH2 domains†

**DOI:** 10.1039/d3sc00746d

**Published:** 2023-05-04

**Authors:** Michelangelo Marasco, John Kirkpatrick, Teresa Carlomagno, Jochen S. Hub, Massimiliano Anselmi

**Affiliations:** a Molecular Pharmacology Program, Memorial Sloan Kettering Cancer Center New York NY USA; b School of Biosciences, University of Birmingham Edgbaston B15 2TT Birmingham UK; c Institute of Cancer and Genomic Sciences, University of Birmingham Edgbaston B15 2TT Birmingham UK; d Theoretical Physics and Center for Biophysics, Saarland University 66123 Saarbrücken Germany massimiliano.anselmi@uni-saarland.de

## Abstract

SHP2 plays an important role in regulating cellular processes, and its pathogenic mutations cause developmental disorders and are linked to cancer. SHP2 is a multidomain protein, comprising two SH2 domains arranged in tandem, a catalytic PTP domain, and a disordered C-terminal tail. SHP2 is activated upon binding two linked phosphopeptides to its SH2 domains, and the peptide orientation and spacing between binding sites are critical for enzymatic activation. For decades, the tandem SH2 has been extensively studied to identify the relative orientation of the two SH2 domains that most effectively binds effectors. So far, neither crystallography nor experiments in solution have provided conclusive results. Using experiment-guided molecular simulations, we determine the heterogeneous structural ensemble of the tandem SH2 in solution in agreement with experimental data from small-angle X-ray scattering and NMR residual dipolar couplings. In the solution ensemble, N-SH2 adopts different orientations and positions relative to C-SH2. We suggest that the intrinsic structural plasticity of the tandem SH2 allows SHP2 to respond to external stimuli and is essential for its functional activity.

## Abstract 2

We have determined the heterogeneous structural ensemble of the tandem SH2 domains of the protein tyrosine phosphatase SHP2 in agreement with experimental data from small-angle X-ray scattering and NMR residual dipolar couplings in solution.

## Graphical abstract

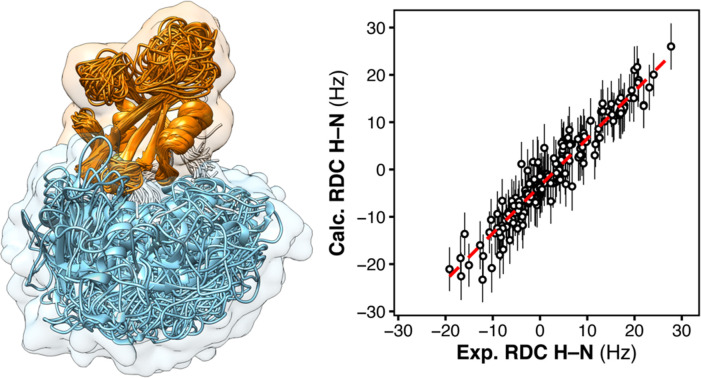

## Introduction

SHP2 (Src-homology 2-containing protein tyrosine phosphatase-2), encoded by the *PTPN11* gene, is an essential regulator of both cell proliferation and immune responses due to its involvement in Ras/MAPK and PD-1 signaling pathways.^1,2^ Unlike most non-receptor protein tyrosine phosphatases, which act as negative regulators of phosphotyrosine cascades, SHP2 increases proliferative signaling by removing inhibitory phosphorylations in the Ras/MAPK pathway and acting as a scaffold protein for Grb2/SOS.^3^ Furthermore, SHP2 is recruited by the immune checkpoint receptor PD-1, which is involved in suppressing T-cell-mediated immune responses, *via* two phosphotyrosine-containing sequences, namely the immune tyrosine inhibitory motif (ITIM), and the immune tyrosine switch motif (ITSM).^4,5^ Consistently with these essential roles, SHP2 has been implicated in several human disorders. Germline SHP2 mutations are found in nearly half of Noonan syndrome patients and in the vast majority of patients with Noonan syndrome with multiple lentigines.^6^ Somatic SHP2 mutations have been also reported in some hematological malignancies, including juvenile myelomonocytic leukemia and, with lower incidence, in B-cell acute lymphoblastic leukemia, acute myeloid leukemia, and myelodysplastic syndrome.^7^ The role of SHP2 mutations in the development of solid tumors is less clear,^2^ but they have been shown to mediate resistance to KRAS^G12C^ and MEK inhibition in several types of cancer.^8,9^ Therefore, SHP2 has become an increasingly important pharmacological target.

From a structural perspective, SHP2 comprises two Src-homology 2 (SH2) domains arranged in tandem (N-SH2 and C-SH2), followed by a protein tyrosine phosphatase (PTP) domain, which hosts the catalytic cysteine (Cys^459^), and a disordered C-terminal tail with two phosphorylation sites (Tyr^542^ and Tyr^580^) separated by a polyproline motif.^10^ The role of the two SH2 domains is to recognize peptide sequences that contain a phosphotyrosine (pY); the nature of the amino acids adjacent to the pY and the specific features of several conserved regions on the surface of the SH2 domains confer specificity to this interaction.^11^ The C-SH2 domain selectively binds phosphopeptides with a single consensus sequence (T/V/I/Y)XpY(A/S/T)X(I/V/L) (where X is any amino acid), whereas N-SH2 is more promiscuous, being capable of binding four different classes of peptides with the following consensus sequences: (I/L/V/M)XpY(T/V/A)X(I/V/L/F), W(M/T/V)pY(Y/R)(I/L)X, (I/V)XpY(L/M/T)Y(A/P/T/S/G), and (I/V/L)XpY(F/M)XP.^12^

Importantly, phosphopeptide binding to the SH2 domains not only permits the recruitment of SHP2 to its correct upstream partners but also regulates its enzymatic activity. The basal catalytic activity of SHP2 is low, because the catalytic site is occluded by an inhibitory autointeraction with the N-SH2 domain.^13^ Oncogenic SHP2 mutations such as SHP2^E76K^ perturb the interaction between N-SH2 and PTP, leading to abnormal constitutive enzymatic activity and uncontrolled cellular proliferation.^14^ In physiological conditions, phosphopeptide binding triggers a conformational change that displaces the N-SH2 domain from the PTP active site and relieves autoinhibition.^10^ Although high-affinity, monovalent phosphopeptides are SHP2 activators by virtue of their binding to N-SH2, bisphosphorylated peptides such as BTAMs (bisphosphoryl tyrosine-based activation motifs) are by far the strongest activators due to their simultaneous interaction with N-SH2 and C-SH2.^15,16^

Although the overall framework of SHP2 activation is relatively well understood, the conformational changes that take place are still a matter of debate. By comparing the crystal structure of SHP2 in the basal, autoinhibited state with the structures of the isolated N-SH2 domain, either bound or unbound to phosphopeptides,^17^ Hof *et al.* first introduced the concept of N-SH2 acting as an allosteric switch.^13^ When bound to the PTP domain, N-SH2 is incapable of binding to phosphopeptides because the EF and BG loops, which line the hydrophobic “specificity pocket”,^11^ are too close to fully accommodate the peptide backbone (the “I state” according to the nomenclature of Hof *et al.*). The structures of the isolated N-SH2 domain differ from the I state adopting the so-called “A state” that is incompatible with PTP binding. Therefore, the N-SH2 domain has two competing ligands, namely the PTP domain and the cognate phosphopeptide, that bind to non-overlapping sites but are nevertheless mutually exclusive. Despite agreeing with biochemical data and explaining why most activating SHP2 mutations cluster at the N-SH2/PTP interface,^18^ this interpretation raises the question of how phosphopeptide binding can occur when N-SH2 is bound to PTP. Furthermore, the role of the EF and BG loops in driving the allosteric switch has recently been questioned. Anselmi and Hub have shown that the closed cleft in the I state is in fact an artifact caused by crystal contacts and that the degree of opening of the cleft has a negligible effect on the free energy of SHP2 activation.^19^ Additionally, the isolated N-SH2 domain bound to a phosphopeptide oscillates between two states, whose principal difference lies in the degree of opening (“unzipping”) of the central β-sheet.^20^ Since the opening of the central β-sheet greatly favors the activation of SHP2, it may represent the main structural determinant of SHP2 activation.^19^ Unzipping of the β-sheet can also be initiated by the binding of weak ligands *via* a dynamic network of correlated motions across the different functional parts of N-SH2.^20,21^ Accordingly, the binding of an isolated pY residue is sufficient to induce a structural rearrangement of N-SH2, provoking an increase in backbone oscillations that lead to the eventual accommodation of the full peptide and SHP2 activation.^21^ Albeit being greatly promoted by pY or phosphopeptide binding, recent evidence has shown that the active state of SHP2, where N-SH2 is detached from PTP, is populated to a small extent even under basal conditions.^22^ This population is increased by pathogenic mutations, such as E76K, and reduced by allosteric SHP2 inhibitors, such as SHP099.^22,23^ Therefore, one of the emerging scenarios is that the high-affinity phosphopeptides may bind to the N-SH2 domain in the minor population of open SHP2, perturbing the motions of its backbone and preventing rebinding of PTP until the peptide dissociates.

Compared to monophosphorylated peptides, the binding of bisphosphorylated peptides induces a larger degree of activation in SHP2 (ref. 15) and is more specific.^16^ The best-known activators of SHP2 in physiological conditions (including IRS1, GAB2, and PD-1) feature two SH2-binding motifs separated by a flexible polypeptide linker. The length of these linkers is approximately 40 Å and this specific value is essential for optimal SHP2 activation; excessive shortening or lengthening of the sequence between two synthetic phosphopeptides leads to a drastic reduction in SHP2 activation.^24^ However, the distance separating two phosphopeptides bound to N-SH2 and C-SH2 in the autoinhibited conformation of SHP2 is significantly larger than 40 Å, being close to 70 Å.^5^ Thus, a large conformational change is needed to bring the two phosphopeptide-binding sites of N-SH2 and C-SH2 into sufficient proximity to allow simultaneous binding of the two pY residues of BTAMs. Accordingly, the formation of a 1:1 complex between the N-SH2–C-SH2 (tandem SH2) domain and a bisphosphorylated peptide has been proposed as a multistep process^25^ that requires overcoming energetic barriers. Finally, the nature of the connection between the two phosphopeptides is also crucial for optimal SHP2 activation. A bisphosphorylated peptide in head-to-tail linkage direction (where the linker connects the C-terminus of the phosphorylated motif *Y̲*′, binding to N-SH2, to the N-terminus of the motif *Y̲*′′, binding to C-SH2, *i.e.*, *Y̲*′–*linker*–*Y̲*′′) is a far better activator than a peptide in tail-to-head linkage direction (with the linker connecting the N-terminus of the phosphorylated motif *Y̲*′ to the C-terminus of the motif *Y̲*′′, *i.e.*, *Y̲*′′–*linker*–*Y̲*′).^24^

Due to its key regulatory role, the tandem SH2 has been extensively studied to identify the relative orientation of the two SH2 domains that most effectively binds effectors and drives activation of SHP2. So far, crystallography has provided contradictory results,^5,24^ while experiments in solutions resulted in conflicting interpretations. Characterizing conformational ensembles of multidomain proteins, such as tandem SH2, poses considerable challenges. Experimental data from either small-angle X-ray scattering (SAXS) or nuclear magnetic resonance (NMR) spectroscopy may be insufficient to define all degrees of freedom of the conformational ensemble, in particular if the ensemble is heterogeneous.^26,27^ Thus, in the literature, SAXS and NMR data have been combined with molecular dynamics (MD) simulations to either derive or validate conformational ensembles of flexible biomolecules, such as intrinsically disordered proteins,^28,29^ protein/RNA complexes,^30^ and multidomain proteins.^31–34^

In this work, we obtained SAXS data and NMR residual dipolar couplings of tandem SH2 in solution and, by means of experiment-guided molecular dynamics simulations, we derived the heterogeneous conformational ensemble of tandem SH2 that is in quantitative agreement with the experimental data. In this ensemble, N-SH2 adopts a plethora of different orientations and positions relative to C-SH2. SHP2 can be recruited by a variety of receptor tyrosine kinases (RTKs), such as PDGFR and FGFR2, as well as by scaffold proteins like Gab-1, FRS2, and IRS, and by PD-1.^35,36^ The BTAMs found within these proteins are all unable to bind to the autoinhibited conformation of SHP2. The linkers separating the phosphotyrosine motifs of the BTAMs have different sequences with a variety of conformational energy landscapes. Thus, we suggest that the intrinsic structural plasticity of tandem SH2 is necessary to enable the activation of SHP2 by a diverse range of upstream partners.

## Results

### In solution, the tandem SH2 adopts a plethora of conformations

**Fig. 1:**
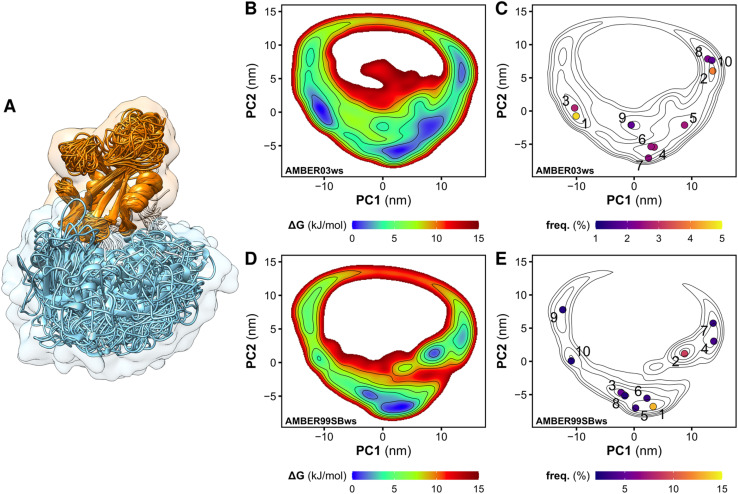
Overlay of the structures of the tandem SH2 obtained from MD simulations. The N-SH2 and C-SH2 domains are depicted as ribbons and colored in cyan and orange, respectively. The structures are superposed at the C-SH2 domain (A). Free energy landscapes along the principal components PC1 and PC2 of the tandem SH2, obtained from the simulation performed with the AMBER03ws force field (B and C) and with the AMBER99SBws force field (D and E). The free energy landscapes are shown as contour plots (isosurface lines indicating steps of 2 kJ mol^−1^) with a color scale (B and D). The projections of the central structures of the ten most populated clusters onto the essential plane are shown in panels C and E. The relative populations of the clusters are indicated by the color scale.

We performed MD simulations of the tandem SH2 domains using the AMBER03ws and AMBER99SBws force fields.^37^ Since certain force fields over-stabilize protein–protein contacts,^37^ force fields with rescaled dispersion interactions between water and protein were used, increasing the solubility of the domains. For each force field, twelve independent simulations of 1 μs each were performed. During the simulations, the tandem SH2 populated a plethora of conformations, with the SH2 domains adopting different relative orientations (Fig. 1A). The flexibility of the linker (residues 105–111) allowed rearrangements of the tandem SH2, either by rotation of the SH2 domains around the principal axis of the tandem SH2 or by bending of the flexible linker. Principal component analysis (PCA) was performed on the Cα atoms of residues Thr^12^–Pro^215^ (*i.e.*, excluding the flexible termini) after least-squares fitting over the Cα atoms. The analysis revealed that interdomain fluctuations largely prevail over intra-domain internal motions, as the first five PCA modes alone encompass almost 90% of the overall fluctuations. We described the relative fluctuations by a minimal set of two modes of motion (represented by the projections PC1 and PC2 onto two essential eigenvectors), which encompassed nearly 60% of the overall tandem SH2 fluctuations. Since the AMBER03ws trajectories showed the most extensive displacements and the highest conformational variability (*i.e.*, the largest sum of eigenvalues), these trajectories were taken as reference and used for the calculation of the principal component eigenvectors. Fig. 1B and D show the free energy landscapes over the essential plane spanned by the two principal components PC1 and PC2, which describe the rotation of the N-SH2 domain relative to C-SH2 (Movie S1†). The free energy landscapes are characterized by a horseshoe shape with at least three energetic basins, roughly corresponding to three regions of the essential plane: (i) a left branch (PC1 < −5 nm), (ii) a central branch (−5 nm < PC1 < 5 nm), and (iii) a right branch (PC1 > 5 nm). The landscapes are characteristic of heterogeneous conformational ensembles.

### The tandem SH2 adopts a prolate shape in solution

**Fig. 2:**
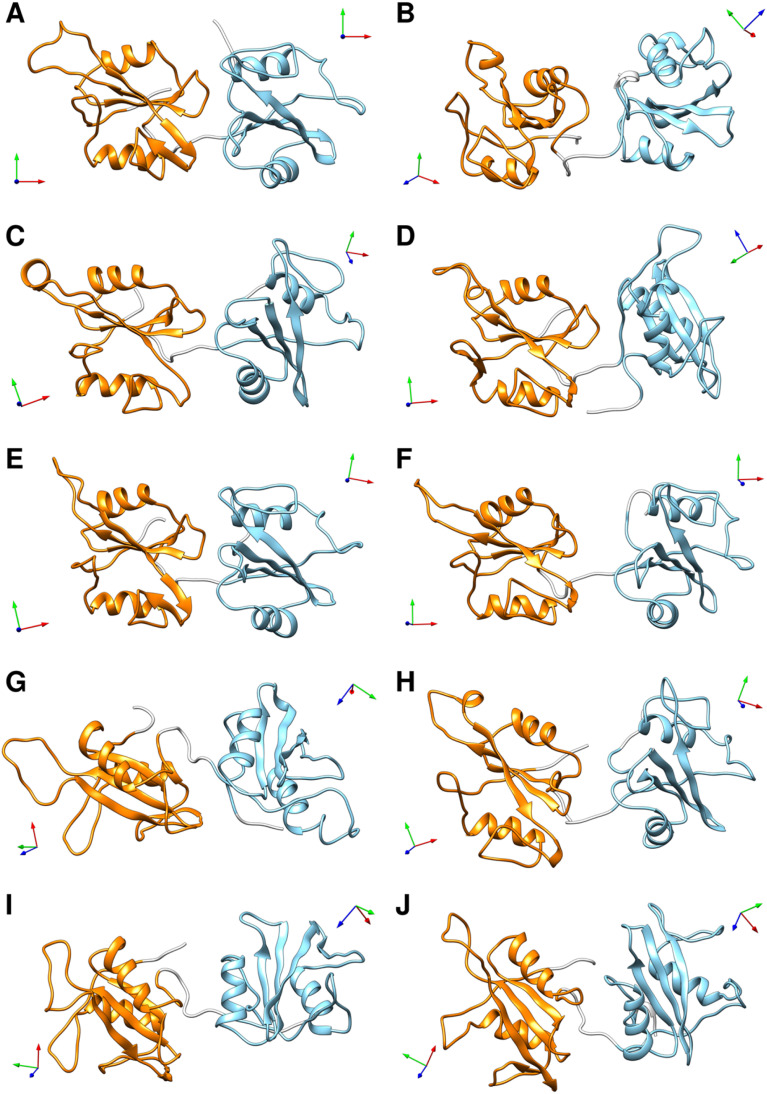
Representative structures of the ten most populated clusters from the MD simulations of the tandem SH2 performed with the AMBER99SBws force field (A–J). The N-SH2 and C-SH2 domains are depicted as ribbons and colored in cyan and orange, respectively. The coordinate axes obtained from the rotation tensors show the rotation of the individual domains relative to the reference structure (in panel A). A representation of the same structures fitted on the C-SH2 domain is given in Fig. S4.†

To select representative molecular structures from the heterogeneous ensemble, cluster analysis was performed on the Cα atoms of residues Thr^12^–Pro^215^ (*i.e.*, excluding the flexible termini). Fig. 1C and E show the central structures of the first ten most populated clusters projected onto the essential plane, which are located near the minima of the free energy landscape. Fig. 2 shows the representative structures of tandem SH2 for the ten most populated clusters obtained with the AMBER99SBws force field. The conformations are characterized by very different relative orientations of the two SH2 domains, as illustrated by the coordinate axes that result from the rotation tensors relative to the reference structure (Fig. 2A). However, these conformations share a similar overall shape. Indeed, during the simulations the shape of the tandem SH2 remains prolate, and the components of the radius of gyration (*R*_gx_ = 1.1 nm, *R*_gy_ = 2.0 nm, *R*_gz_ = 2.0 nm) are virtually constant (Fig. S1†). These results suggest that the different conformations of the tandem SH2 cannot be distinguished by small-angle X-ray scattering experiments since they would give rise to similar SAXS curves.

### The flexible linker confers structural plasticity and functional adaptability to the tandem SH2

**Fig. 3:**
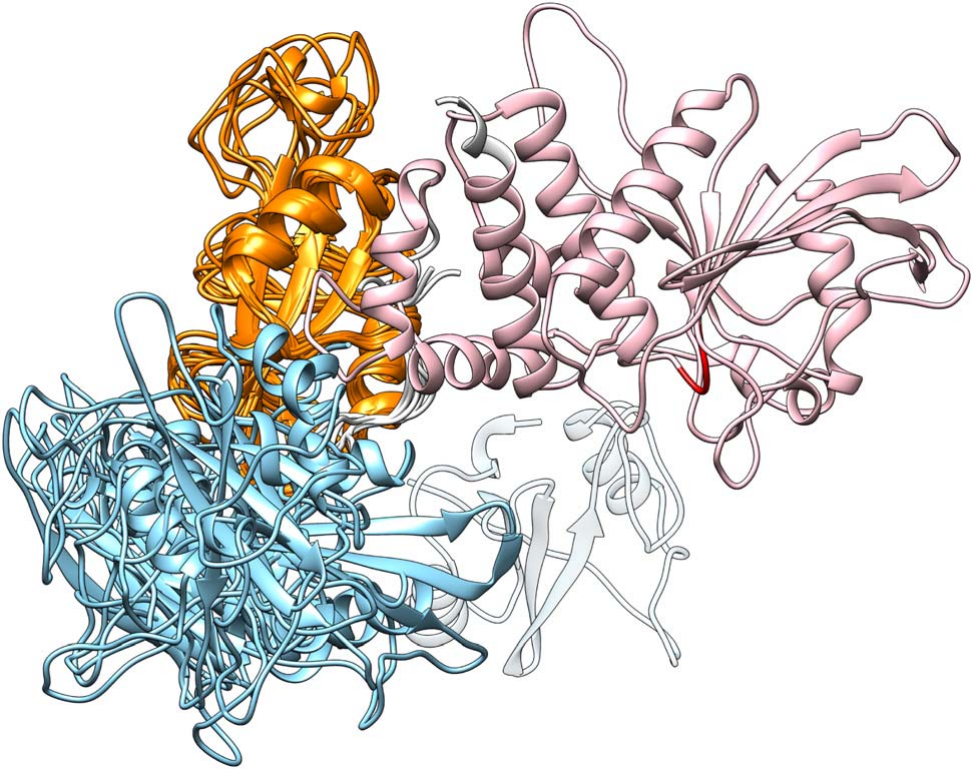
Overlay at the C-SH2 domain (colored in orange) of the autoinhibited structure of SHP2 and of the representative structures of the clusters 1 and 3–8, which did not exhibit significant spatial clashes between N-SH2 (cyan) and PTP (pink). The N-SH2 domain in the autoinhibited structure is shown as transparent ribbon. The catalytic loop of PTP is indicated in red.

Although the overall shape of the tandem SH2 did not vary significantly, the two domains underwent large rearrangements of their positions relative to one another, as evidenced by superposing the conformations using the C-SH2 domain as reference (*cf.*Fig. 1A). The internal fluctuations of the individual domains (Fig. S2†) were not correlated with each other, suggesting that the internal dynamics of the two SH2 domains are largely independent from one another (Fig. S3†). The central structures of the ten most populated clusters from the AMBER99SBws trajectories (Fig. S4,†*cf.*Fig. 2) are stabilized by salt bridges between Glu^139^ and Arg^4^–Arg^5^ and by several first-order water-mediated hydrogen bonds (Table S1†) between the N-SH2 domain (residues 3–104) and the C-SH2 domain (residues 112–216). These central structures significantly deviate from the conformation adopted by the tandem SH2 in the autoinhibited state of SHP2 (*i.e.*, the “closed” conformation, Fig. S5†). Furthermore, with the exception of clusters 2, 9 and 10, the central structures would be compatible with active conformations of SHP2 where the N-SH2 domain has moved away from the PTP domain (*i.e.*, the open conformations), since no significant spatial clashes would arise between N-SH2 and PTP if the structures of tandem SH2 were superposed over the autoinhibited structure of SHP2 at the C-SH2 domain (Fig. 3). This observation suggests that the conformational space of tandem SH2 might be critical to resolve the ensemble of active SHP2 in a future study.

In closing, the flexible linker enables high structural plasticity of the tandem SH2, allowing N-SH2 to adopt different orientations and positions relative to C-SH2. This plasticity is critical for the function of SHP2. Indeed, both the position and the orientation of N-SH2 in the inactive state of SHP2, which are dictated by the high-affinity interaction with PTP, do not allow the binding of BTAM. To achieve SHP2 activation, N-SH2 needs to initially dissociate from the catalytic site and adopt orientations relative to C-SH2 that satisfy the spatial constraint imposed by the BTAM chain length. Thus, the flexible linker and the structural variability of the tandem SH2 provide the adaptability necessary for responding to external stimuli and allow SHP2 to perform its function.

### In solution, the tandem SH2 adopts conformations compatible with the binding of BTAMs

**Fig. 4:**
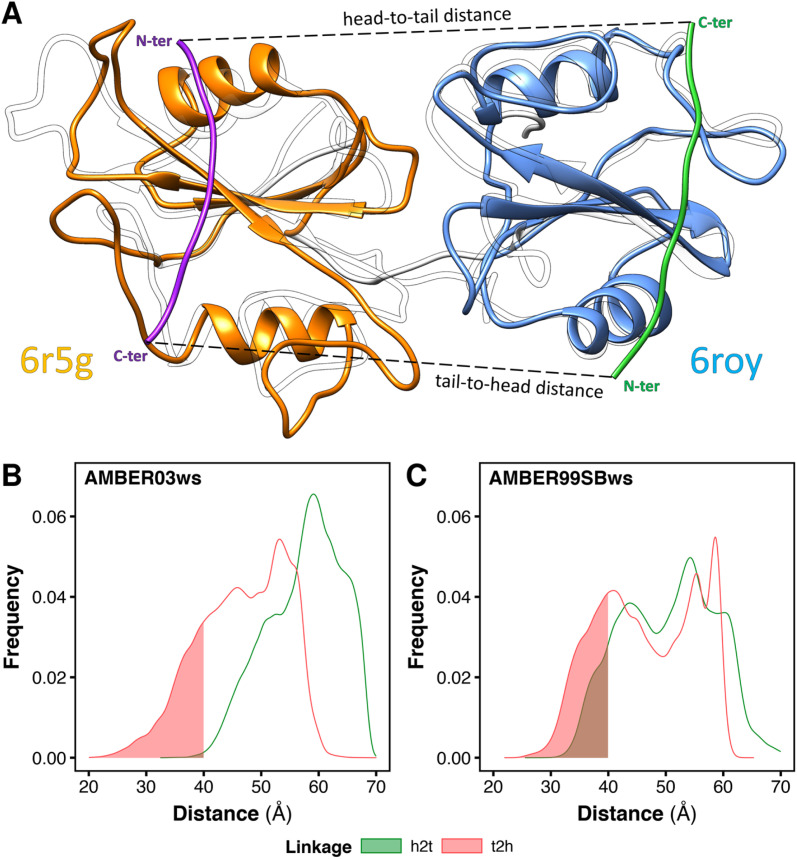
Minimum head-to-tail (*h2t*) and tail-to-head (*t2h*) distances for the conformations adopted by the tandem SH2 during the MD simulations. The distances were determined by superposing respectively the structures of N-SH2 bound to the ITIM peptide (PDB ID 6roy) and of C-SH2 bound to the ITSM peptide (PDB ID 6r5g) on every tandem SH2 structure from MD simulations (A). Distributions of the *h2t* and *t2h* distances as obtained from the simulations performed with the AMBER03ws force field (B) and with the AMBER99SBws force field (C), respectively. The intervals of the distributions with distances below 40 Å are shaded.

In principle, the linkage between the two SH2-binding motifs of bisphosphorylated peptides may occur in two distinct directions (Fig. 4A): (i) from the C-terminus of the peptide binding the N-SH2 domain toward the N-terminus of the peptide binding the C-SH2 domain (termed head-to-tail, *h2t*), (ii) or *vice versa*, from the C-terminus of the peptide binding the C-SH2 domain toward the N-terminus of the peptide binding the N-SH2 domain (termed tail-to-head, *t2h*). Although peptide–SH2 interactions are identical for the two linkage directions (Fig. 4A, dashed lines indicate the linker between the motifs), the two complexes resulting from *h2t* and *t2h* linkages are topologically different.

The natural sequences of BTAMs mainly adopt the *h2t* direction, and experimental activation assays of SHP2 have shown that *h2t* BTAMs are more effective activators than their *t2h* counterparts.^24^ Moreover, the linkage distance is functionally relevant since a linkage distance of ∼40 Å triggers the most effective enzymatic activation of SHP2.^24^

To test whether the conformations of the tandem SH2 generated by MD simulations are compatible with either the *h2t* or the *t2h* linkage direction (or with both of them), we estimated the minimum head-to-tail and tail-to-head distances for each conformation adopted by the tandem SH2 in solution. The calculation was performed superposing the structures of N-SH2 bound to the ITIM peptide (PDB ID 6roy)^5^ and of C-SH2 bound to the ITSM peptide (PDB ID 6r5g)^5^ on the tandem SH2 (Fig. 4A). This procedure was repeated for each frame of the trajectories, and the *h2t* distance was calculated as the C–N distance between the residue in position +6 of ITIM (Gln^6^) and the residue in position −4 of ITSM (Glu^−4^), whereas the *t2h* distance was calculated as the C–N distance between the residue in position +6 of ITSM (Pro^6^) and the residue in position −4 of ITIM (Phe^−4^). Fig. 4B and C report the distributions of the *h2t* and *t2h* distances as obtained from the simulations performed with the AMBER03ws and with the AMBER99SBws force field, respectively. 11% of the tandem SH2 conformations obtained with the AMBER99SBws force field had a head-to-tail distance shorter than 40 Å, while only 0.2% of the conformations obtained from the AMBER03ws force field satisfied this condition. Thus, only the structures obtained with AMBER99SBws are compatible with the optimal linkage distance of 40 Å. Interestingly, both the AMBER03ws and the AMBER99SBws force fields provided a large fraction of structures with a tail-to-head distance shorter than 40 Å (21% and 27%, respectively). These results suggest that the weaker activation of SHP2 by tail-to-head BTAMs is not due to the physical restraints imposed by the tandem SH2 on this connectivity, but may instead arise from other factors.^24^

### Current crystal structures of the *PTPN11* tandem SHP2 are not compatible with the binding of BTAMs

**Fig. 5:**
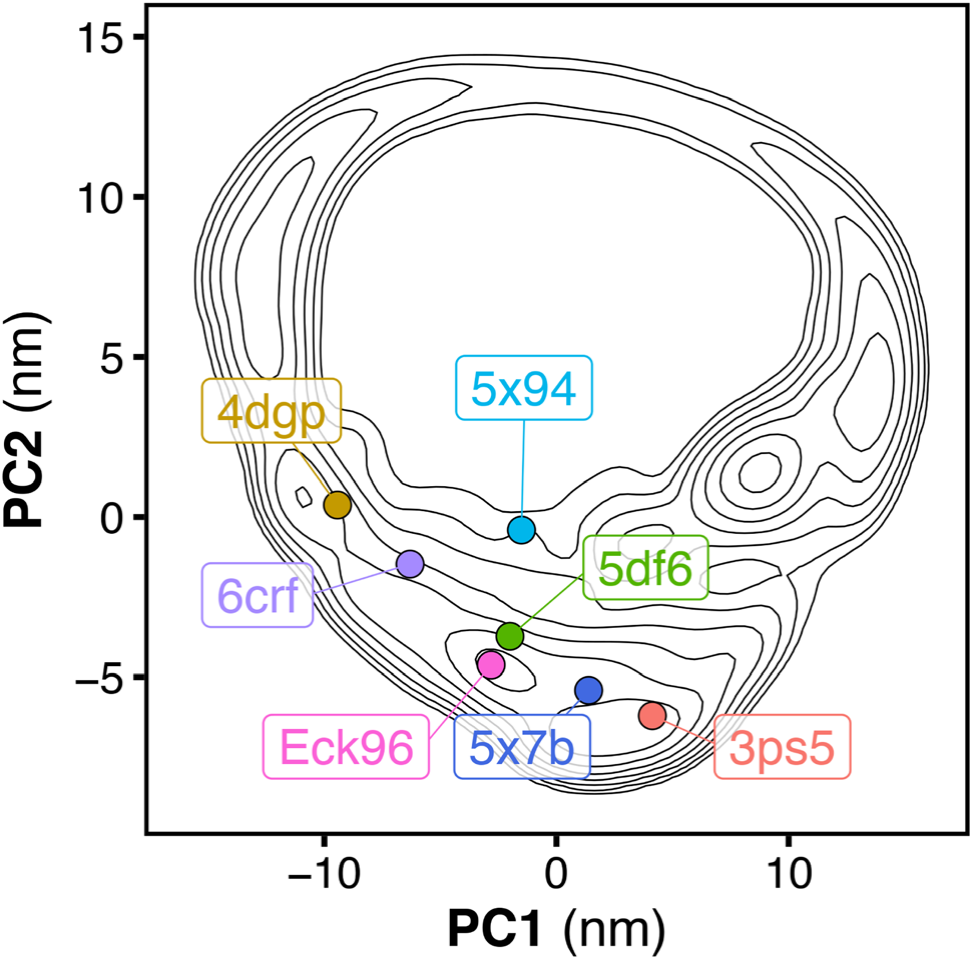
Free energy landscape along the principal components PC1 and PC2 of the tandem SH2, obtained from the simulation performed with the AMBER99SBws force field. The free energy landscape is presented as contour plot (isosurface lines indicating steps of 2 kJ mol^−1^). The dots represent the projections onto the essential plane of the following crystal structures of the tandem SH2: SHP2 in autoinhibited, closed state (4dgp, gold), the leukemia-associated SHP2^E76K^ mutant in active, open state (6crf, violet), the *PTPN11* tandem SH2 in complex with phosphopeptides (5df6, green; Eck96, purple; 5x94, cyan; 5x7b, blue), SHP1 in active, open state (3ps5, red).

Fig. 5 shows tandem SH2 conformations in a variety of currently available crystal structures projected onto the essential plane spanned by the principal components PC1 and PC2. The crystal structures include: (i) SHP2 in an autoinhibited, closed state (4dgp),^38^ (ii) the leukemia-associated SHP2^E76K^ mutant in an active, open state (6crf),^39^ (iii) the *PTPN11* tandem SH2 in complex with phosphopeptides (5df6, Eck96, 5x7b, 5x94),^24,40,41^ (iv) SHP1 (the closest homolog of SHP2) in an active, open state (3ps5).^42^ The crystal structures were determined under different experimental conditions and within different space groups, which resulted in different arrangements of the tandem SH2 (Fig. 5, Table 1). While all conformations observed in the crystal structures were sampled during our simulations, not all possible arrangements of the tandem SH2 observed in our simulations have been found in crystals. In addition, the two principal components determined by PCA analysis of the MD simulations distinguished well the different crystal structures.

**Table 1:** Parameters of crystal structures containing the tandem SH2 domains of SHP2 and SHP1 proteins

PDB ID	Head-to-tail distance Å	Tandem SH2/linker clashes?	Space group	Description
4dgp	65.8	Yes	*P*2_1_2_1_2	SHP2 wt closed
6crf	64.4	Yes	*C*121	SHP2 E76K open
5df6	61.3	Yes	*P*12_1_1	*PTPN11* tandem SH2
Eck96	56.5	Yes	*P*2_1_	*PTPN11* tandem SH2
5x7b	57.5	Yes	*P*2_1_2_1_2	*PTPN11* tandem SH2
5x94	68.5	Yes	*P*12_1_1	*PTPN11* tandem SH2
3ps5	38.4	No	*H*32	SHP1 wt open

As previously reported,^5^ the relative orientations of the SH2 domains in both the autoinhibited structure of wild-type SHP2 (PDB ID 4dgp)^38^ and the open structure of the constitutively active SHP2^E76K^ mutant (PDB ID 6crf)^39^ are incompatible with simultaneous binding of the two phosphorylated motifs of a BTAM to both SH2 domains, due to the spatial constraint imposed by the 40 Å linker (Table 1). The same is true for crystal structures of the tandem SH2 in complex with phosphopeptides (PDB IDs 5df6,^40^5x7b,^41^5x94,^41^ and Eck96 (ref. 24) (structure not deposited) with head-to-tail distances of approximately 61 Å, 58 Å, 69 Å, and 53 Å, respectively). In contrast, the open conformation of SHP1 (PDB ID 3ps5)^42^ could be compatible with binding of BTAMs since (i) the head-to-tail distance is 38.4 Å and thus shorter than the maximum linker extension of 40 Å and (ii) the structure would not impose steric clashes between the tandem SH2 and the peptide linker (Table 1). Overall, the tandem SH2 arrangement found in the open SHP1 structure is currently the only crystallography-derived arrangement of tandem SH2 that could be bound by an activating BTAM.

### The heterogeneous solution ensemble of the tandem SH2 reproduces the experimental SAXS curve

**Fig. 6:**
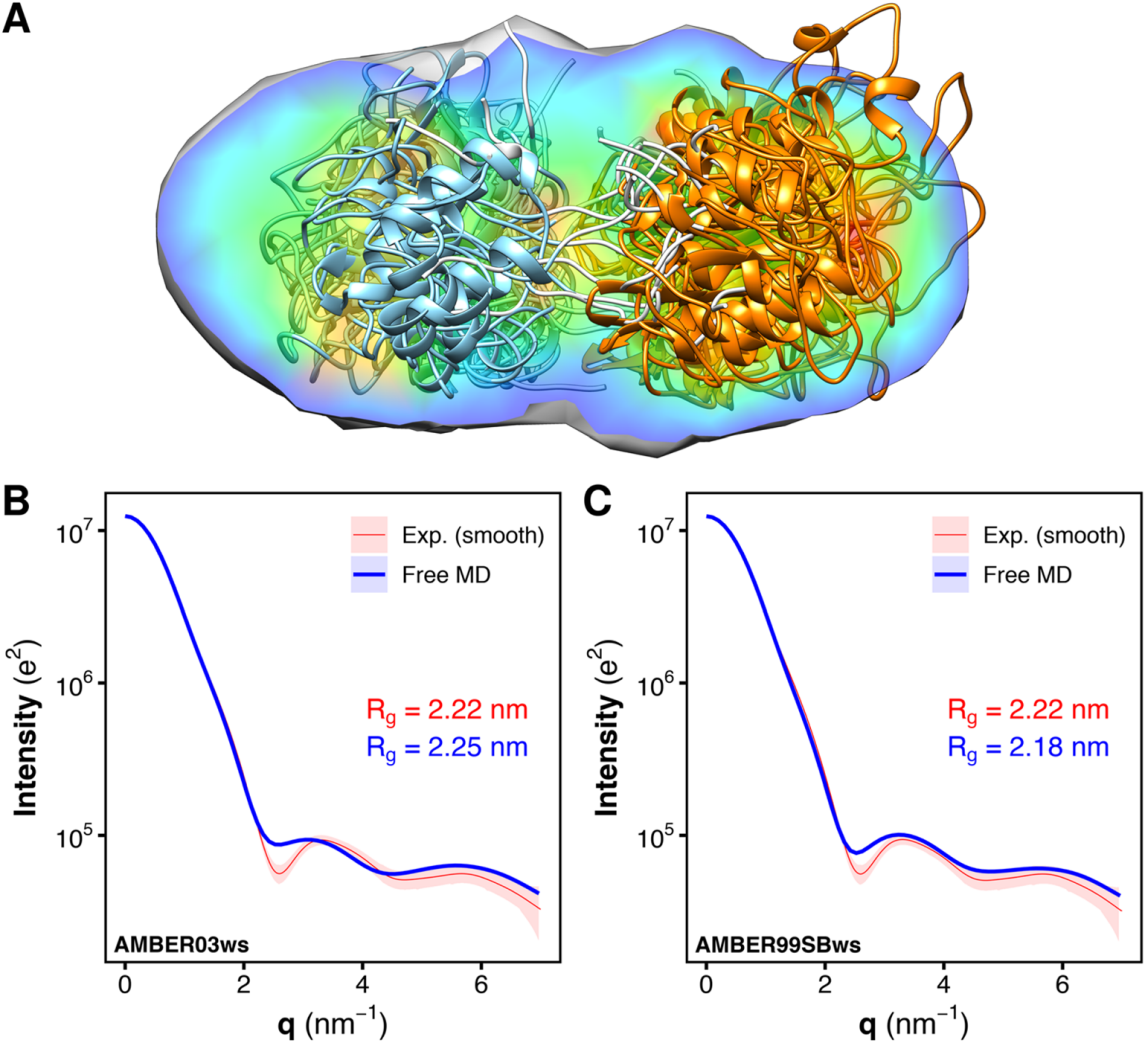
Overlay of the electron density reconstruction from the experimental solution scattering data with the representative structures of the tandem SH2 shown in Fig. 2. The electron density reconstructed from the SAXS curve is shown as volume colored according to density. The representative structures are depicted as ribbons. N-SH2 and C-SH2 domains are colored in cyan and orange, respectively (A). Comparison of the experimental small-angle X-ray scattering (SAXS) curve (red), reported as a smoothed curve, with the calculated SAXS curves (blue) of the solution ensemble of the tandem SH2, obtained from MD simulations with the AMBER03ws (B) and the AMBER99SBws force field (C), respectively. Experimental and calculated radii of gyration (*R*_g_) are reported in each panel.

To validate the MD-derived solution ensemble, we collected SAXS curves of tandem SH2 in solution. Since the three-dimensional electron density reconstruction, calculated by DENSS^43^ from the experimental solution scattering data (Fig. 6A), provided only moderate agreement (averaged correlation = 0.68, *χ*^2^ = 13.9) and ambiguous overlapping with the ensemble of the ten representative structures shown in Fig. 2, we used explicit-solvent SAXS calculations^44^ to predict SAXS curves from the trajectories obtained from the unrestrained, “free” MD simulations performed using the AMBER03ws and AMBER99SBws force fields (Fig. 6B and C). The calculated SAXS curves were compared with both the raw data (Fig. S6†) and the smoothed curve obtained by ATSAS (Fig. 6B and C).^45^ The AMBER03ws force field provides a better agreement at lower values of *q* (less than 2 nm^−1^), whereas the AMBER99SBws force field better reproduces the SAXS curve features over the entire range of *q*. The AMBER03ws and AMBER99SBws trajectories returned values of the radius of gyration of 2.25 and 2.18 nm, respectively, which are both close to the experimental value of 2.22 nm.

SAXS curves were also calculated individually for the ensembles corresponding to the ten most populated clusters from the AMBER03ws and the AMBER99SBws trajectories (Fig. S7†). Although the AMBER03ws force field consistently yielded curves and radii of gyration in reasonable agreement with the experiments (Fig. S7A–J†), along with rather low values of *χ*^2^ (Fig. S8A†), the force field did not reproduce the finer features of the experimental SAXS curve, in particular the depth of the first minimum at *q* = 2.6 nm^−1^. Instead, the AMBER99SBws force field was able to reproduce all key features of the experimental SAXS curve in some cases, with a perfect match for clusters 3 and 9 (Fig. S7K–T†), but also returned some conformations with high *χ*^2^ (Fig. S8B†), specially for clusters 4 and 5. Notably, the SAXS curves obtained from distinct clusters were very similar, showing that the tandem SH2 may populate a heterogeneous conformational ensemble in solution, yet retaining a similar shape.

The analysis of the SAXS curves suggested that both the AMBER03ws and the AMBER99SBws force fields are capable of reproducing the experimental SAXS curve with a good approximation. However, only AMBER99SBws provided conformations capable of binding BTAM, reconciling both the structural and functional features of the tandem SH2.

### Comparison of residual dipolar couplings from NMR with free MD simulations confirms the existence of a heterogeneous conformational ensemble in solution

To provide further experimental evidence of a heterogeneous structural ensemble of tandem SH2, we measured NMR residual dipolar couplings (RDCs), and then investigated the agreement between this set of experimental RDCs and the corresponding sets of back-calculated RDCs derived either using single structures of the unrestrained ensemble or using sub-ensembles selected therefrom. Briefly, back-calculation of RDCs based on a template structure or a set of structures involves an initial step of fitting the experimental RDCs to the template structure(s) *via* singular value decomposition (SVD)^46^ to obtain an alignment tensor (or tensors); the resulting tensor(s) can then be used in combination with the template structure(s) to compute the set of back-calculated RDCs. In the context of a set of multiple template structures, three approaches can be envisaged: (i) each template structure can be fitted separately to the experimental RDCs, yielding a single alignment tensor and a corresponding set of back-calculated RDCs per structure, (ii) the set of template structures can be fitted simultaneously to the experimental RDCs in such a way as to yield a single alignment tensor and an ensemble-averaged set of back-calculated RDCs, and (iii) the set of template structures can be fitted simultaneously to the experimental RDCs to yield a corresponding set of alignment tensors (one tensor per template structure).^47^ The results from the third approach (multiple-template/multiple-tensor) using 20 structures randomly selected from the AMBER99SBws structural ensemble are presented in Fig. S9.† The correlation is satisfactory for H–N RDCs, but the other three sets of RDCs (N–C′, H–C′ and C′–Cα) show clear outliers. Fig. S10† compares the performance of the three approaches (single-template/single-tensor, multiple-template/single-tensor and multiple-template/multiple-tensor) using two types of sub-ensembles of template structures selected from the full unrestrained MD ensemble: (i) a population-weighted set of central structures from the largest clusters, or (ii) an unweighted set of structures randomly chosen from the MD-derived ensemble. It should be noted that the multiple-template/single-tensor approach is only valid when all members of the experimental ensemble exhibit the same alignment tensor, but this criterion is difficult to test without simulating tensors from the template structures *a priori*. In our case, the similarity of the overall molecular shape of the structures of the MD ensemble suggested that a corresponding similarity of the respective alignment tensors was a plausible possibility. Firstly, as expected, the single-structure/single-tensor approach yields poor Pearson correlation coefficients (<0.65) between the experimental and back-calculated RDCs, which depend strongly on the randomly selected structure. The multiple-structure/single-tensor approach yields correlation coefficients that plateau at ∼0.65 and ∼0.70 for the two types of sub-ensembles, respectively, but only the multiple-structure/multiple-tensor approach reaches correlation coefficients of ∼0.9, indicating that the assumption of a single invariant alignment tensor across the ensemble is not valid. Overall, these results support the conformational heterogeneity of the tandem SH2 in solution, in agreement with the heterogeneity exhibited by the MD-derived ensemble.

### Ensemble refinement against experimental RDCs further supports a heterogeneous ensemble

For each individual structural conformer, the residual dipolar coupling between two nuclei depends on the value of 〈3 cos^2^ *ϑ* − 1〉, where *ϑ* is the angle between the internuclear vector and the external magnetic field for a given molecular orientation, and the average runs over the macroscopic ensemble of all possible molecular orientations in solution. An alignment medium induces an anisotropic orientation distribution, over which 〈3 cos^2^ *ϑ* − 1〉 ≠ 0 for a general internuclear vector. In the presence of conformational heterogeneity, each conformer would be associated with a corresponding macroscopic ensemble and an anisotropic orientation distribution thereof. However, if structural interconversion between different conformers is sufficiently rapid on the RDC timescale (approximately the inverse of the magnitudes of the RDCs), only a single set of RDCs is experimentally observable, corresponding to the ensemble-average of the sets of RDCs that would arise from the individual structural conformers.

**Fig. 7:**
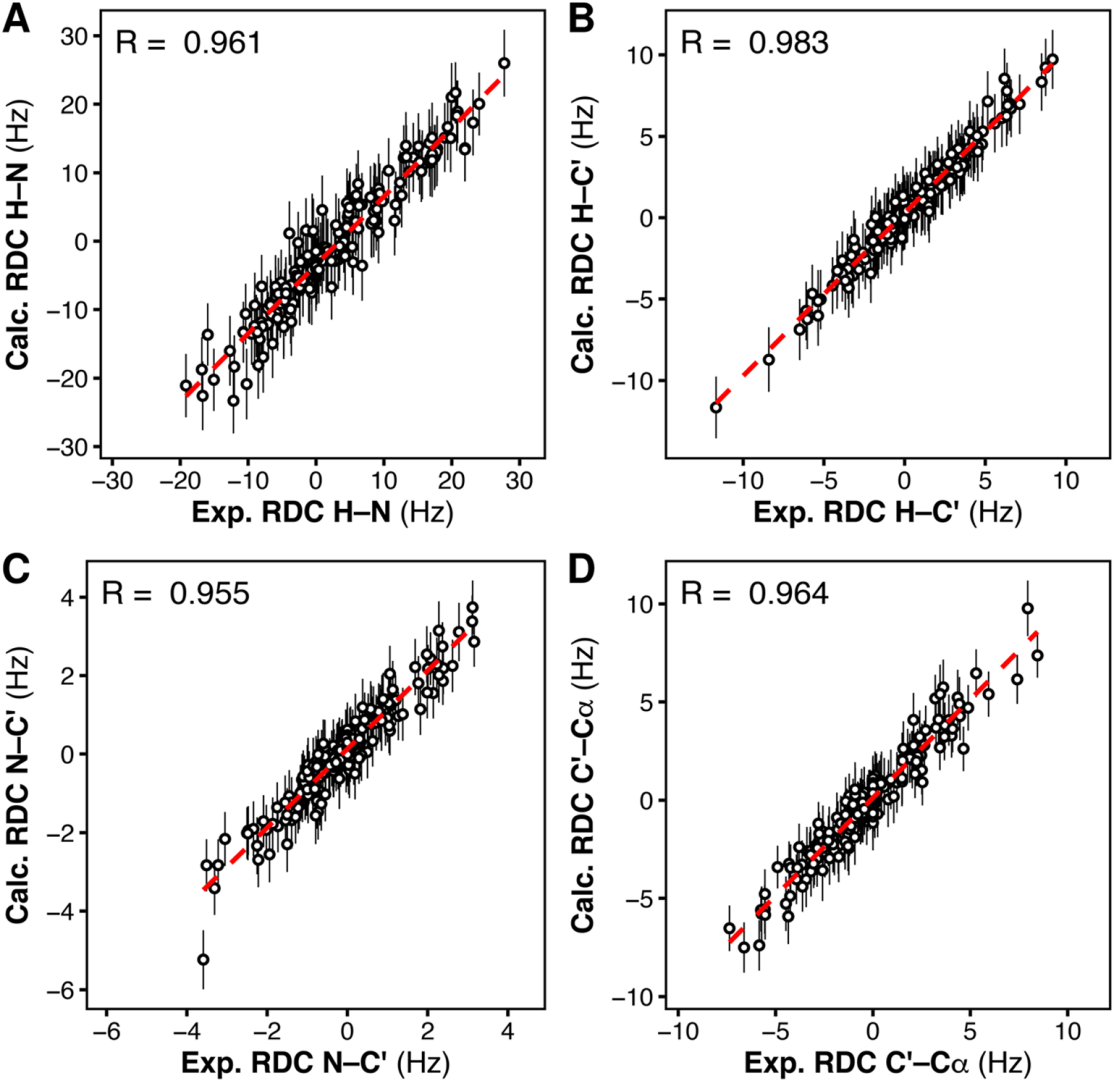
Correlation between the experimental residual dipolar couplings (exp. RDC) and the calculated residual dipolar couplings (calc. RDC), for each type of RDC, H–N (A), H–C′ (B), N–C′ (C), C′–Cα (D), as obtained from RDC-restrained multi-replica MD simulations of the tandem SH2, starting from 24 conformations representing the complete solution ensemble.

To reproduce this scenario *in silico* and find the structural ensemble that best reproduces the RDC data, we performed RDC-restrained MD simulations. For every pair of nuclei for which an RDC was experimentally available, we introduced a structural restraint to minimize the deviation between (i) the angle *ϑ* derived from the experimental RDC and (ii) the angle *ϑ* calculated on-the-fly from the RDC-restrained MD simulation.^48^ To account for the heterogeneity of the structural ensemble, RDCs were calculated from a parallel-replica MD simulation of the tandem SH2 using 24 replicas and the AMBER99SBws force field. The starting coordinates were taken from random members of the heterogeneous, unrestrained ensemble. The correlation between the RDCs calculated from the final RDC-refined ensemble and the experimental RDCs was higher than 0.9, demonstrating good agreement of the simulated heterogeneous ensemble with our experimental data (Fig. 7).

To exclude that RDC restraints lead to overfitting of the refined ensemble, which would be manifested as physically unrealistic local geometries, we compared the structures of the individual N-SH2 and C-SH2 domains in the RDC-restrained ensemble with those of the unrestrained MD ensemble. Fig. S11A† reports the distribution of RMSDs from the respective reference structures (PDB ID 5df6)^40^ for the N-SH2 domain and the C-SH2 domain belonging to the tandem SH2. The deviations from the reference structures in the RDC-restrained ensemble were comparable to those observed in the unrestrained ensemble. These findings demonstrate that the restraints introduced to reach an agreement with the experimental RDCs did not perturb the domain structures, suggesting that the unrestrained ensemble is a reasonable representation of the experimental ensemble.

To verify whether the internal dynamics of individual domain were influenced by the RDC restraints, we compared the distribution of the pairwise RMSD values across the structures of the RDC-restrained ensemble with the corresponding RMSD distribution of the unrestrained ensemble (Fig. S11B†). The respective RMSD distributions were similar (Fig. S11B†), indicating that the dynamics of the individual SH2 domains remained mostly unperturbed during the RDC-restrained simulations.

As a negative control, we also tested whether ensembles with more homogeneous interdomain orientations could reproduce the experimental RDCs and the SAXS curve. We performed RDC-restrained, replica-averaged MD simulations starting from conformations belonging either to the ensemble of the most populated cluster 1 or to the ensembles of clusters 3 and 9, which provided the best agreement with the experimental SAXS curve (*cf.* Fig. S7†). All the cluster-specific ensembles provided correlations between the calculated and the experimental RDCs of similar quality as those yielded by the complete MD-derived ensemble (Fig. S12–S14†). Also, the calculated SAXS curves (Fig. S15†) were substantially unchanged compared to those obtained from the respective unrestrained cluster-specific ensembles: the ensembles from cluster 3 and 9 maintained an excellent agreement with experimental SAXS curve (Fig. S15B and C†), while the RDC-restrained ensemble from cluster 1 (Fig. S15A†) underestimated the radius of gyration (*R*_g_ = 2.13 nm), as did the unrestrained ensemble of cluster 1. However, during the RDC-restrained simulations, only the ensembles from clusters 1 and 3 remained homogeneous, while the ensemble starting from cluster 9 was not stable and adopted other interdomain orientations. In addition, both the structure and the structural variability of the individual domains were substantially perturbed in the RDC-restrained ensembles from cluster 1, 3, and 9 (Fig. S11†). We conclude that only the structural ensemble of tandem SH2 containing diverse interdomain orientations (as in the full MD-derived ensemble) was able to provide an excellent correlation with the experimental RDC and SAXS data, without perturbing the native fold and the structural variability of the individual domains.

In conclusion, RDC data could be reproduced with an ensemble of conformations that preserves the structure and dynamics of individual domains observed in the unrestrained MD simulations if – and only if – the ensemble contains a plethora of diverse interdomain orientations. The RDC-refined ensemble also provides a good fit to the experimental SAXS curve (Fig. S16†). Overall, these results prove that the variability in interdomain orientation observed in the unrestrained MD-derived ensemble provides good agreement with both RDC and SAXS data.

### The binding of head-to-tail BTAMs is coupled with the maximum displacement of N-SH2 from PTP

Experimental activation assays of SHP2 demonstrated that head-to-tail BTAMs are more effective activators than their tail-to-head counterparts.^24^ Originally, Eck *et al.* suggested that this might be due to the PTP domain, whose presence might interfere with the binding of tail-to-head linked peptides.^24^ To gain more insight into the origin of this asymmetry, we studied how the head-to-tail and tail-to-head distances correlate with PTP blockage by the N-SH2 domain. Besides the *h2t* and *t2h* distances, we considered also the center-of-mass distance (*d*_N-SH2_) and the root mean squared deviation (*rmsd*) between the N-SH2 domains in the tandem SH2 and in autoinhibited SHP2 (PDB ID 4dgp),^38^ calculated after superposition of the two structures at the C-SH2 domain.

Properties of the head-to-tail (*h2t*) and of the tail-to-head (*t2h*) distances, *d*_h2t_ and *d*_t2h_, are reported for the AMBER99SBws ensemble in Fig. S17† as functions of PC1 and PC2 in the essential plane. These properties include: (i) the smallest values, min(*d*_h2t_) and min(*d*_t2h_), (ii) the average values, 
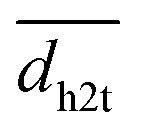
 and 
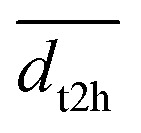
, (iii) and the largest values, max(*d*_h2t_) and max(*d*_t2h_). The three statistical features suggest that the short *h2t* distances are mostly located in the central-right branch of the essential plane. In contrast, the short *t2h* distances are mostly found in the central-left branch. These results suggest that only the configurations gathering at the central branch of the essential plane would bind BTAMs with both *h2t* and *t2h* linkage. In all other cases, due to the rotation of the N-SH2 domain relative to C-SH2, as captured by PC1 and PC2, a decrease of the *h2t* distance is often coupled with an increase of the *t2h* distance, or *vice versa*.

Analogously, Fig. S18† reports the center-of-mass distance (*d*_N-SH2_) and the root mean squared deviation (*rmsd*) as functions of PC1 and PC2. Small *d*_N-SH2_ distances are mostly found at intermediate PC2 values (0 < PC2 < 5). The largest values of *d*_N-SH2_ are located at the vertex of the horseshoe distribution, in the same region as the projection of the crystal structure of SHP1 (*cf.*Fig. 5). However, the values of *rmsd* tend to be smaller in the left branch of the distribution. These results suggest that though the conformations clustering at both the left and right branches might be close to the PTP active site, the conformations clustering at the right branch could not bind PTP due to a flipped orientation of N-SH2. In fact, the crystal structure of the autoinhibited SHP2 is found in the left branch (*cf.*Fig. 5).

Taken together, these results suggest that some of the conformations that are able to bind BTAM with *t2h* linkage would still allow N-SH2 to be near the active site of PTP. On the other hand, the peptides with *h2t* linkage tend to select for conformations of N-SH2 that are drastically different from the autoinhibited structure of SHP2 (Fig. S19 and S20†). This observation partially rationalizes why BTAMs with *h2t* linkage would lead to stronger activation of SHP2 than their *t2h* counterparts.^24^ However, further investigations in the presence of the full-length SHP2 will be needed to fully clarify the significance of the two linkage directions.

## Conclusions

We combined SAXS, NMR spectroscopy and experiment-guided MD simulations to show that the tandem SH2 adopts in solution a plethora of distinct conformations. Although the characteristic features of the experimental SAXS curve could in principle be compatible with a single conformation adopted by the tandem SH2, deeper analysis revealed that the wide range of solution conformations share a similar prolate shape and size, thereby giving rise similar SAXS profiles. Moreover, only such a heterogeneous ensemble reproduced the experimental RDCs from NMR without perturbing the native domain fold and dynamics. The AMBER99SBws force field best reproduced all structural and functional features of the tandem SH2, demonstrating its suitability for future computational studies on SHP2. Finally, we showed that binding of head-to-tail BTAMs to the tandem SH2 is coupled with maximum displacement of N-SH2 from PTP, suggesting a mechanism by which head-to-tail BTAMs are more effective activators than their tail-to-head counterparts.

## Data availability

Experimental and computational data is deposited in the ESI.† Additional analysis of molecular dynamics trajectories; comparison between the experimental and the calculated SAXS curves; comparison between the experimental and the calculated residual dipolar couplings; Movie S1† showing the rotation of the N-SH2 domain relative to C-SH2 as described by principal components PC1 and PC2.

## Author contributions

M. M. prepared samples. M. M. and J. K. recorded and analyzed SAXS and NMR data. T. C. designed and supervised SAXS and NMR experiments. M. A. designed, supervised and performed MD simulations, analysis and comparison with experimental data. M. A. and J. S. H. designed and supervised SAXS-refined calculations. M. A. and M. M. drafted the manuscript. All authors wrote the final version of the manuscript.

## Conflicts of interest

The authors declare no conflicts of interest.

## Supplementary Material

SC-014-D3SC00746D-s001

SC-014-D3SC00746D-s002
