# Supplementary material for: Experiment-guided molecular simulations define a heterogeneous structural ensemble for the PTPN11 tandem SH2 domains
Source: Chem Sci. 2023 May 4;14(21):5743–55. doi: 10.1039/d3sc00746d (PMC10231330; doi:10.1039/d3sc00746d)
Supplement: SC-014-D3SC00746D-s001 [file SC-014-D3SC00746D-s001.pdf]

Electronic Supplementary Material (ESI) for Chemical Science.
This journal is © The Royal Society of Chemistry 2023

Supplementary Information

Experiment-guided molecular simulations define a heterogeneous structural ensemble for the *PTPN11* tandem SH2 domains

Michelangelo Marasco,[1] John Kirkpatrick,[2] Teresa Carlomagno,[2,3] Jochen S. Hub,[4]

Massimiliano Anselmi[4,]*

[1]Molecular Pharmacology Program, Memorial Sloan Kettering Cancer Center, New York, NY, USA

[2]School of Biosciences, University of Birmingham, Edgbaston, B15 2TT, Birmingham, UK

[3]Institute of Cancer and Genomic Sciences, University of Birmingham, Edgbaston, B15 2TT, Birmingham, UK

[4]Theoretical Physics and Center for Biophysics, Saarland University, 66123 Saarbrücken, Germany

This PDF file includes:
Table S1
Figures S1 to S20
Methods

Other supplementary materials for this manuscript include the following:
Movie S1

Table S1. First-order water-mediated H-bonds between the SH2 domains of tandem SH2 calculated for each cluster obtained with the AMBER99SBws force field.

# Cluster	N-SH2 residue	C-SH2 residue	Occurrence (% of time)
1	Arg^{5}	Glu^{139}	88
	Tyr^{63}	Ser^{142}	35
	Arg^{4}	Glu^{139}	30
2	Tyr^{100}	Glu^{121}	88
	Arg^{5}	Glu^{139}	81
	Lys^{99}	Glu^{121}	48
3	Arg^{5}	Glu^{139}	94
	Arg^{5}	Pro^{144}	75
	Asn^{10}	Asp^{146}	48
	Phe^{7}	His^{143}	36
4	Arg^{5}	Glu^{139}	100
	Pro^{101}	His^{116}	54
	Tyr^{100}	Gln^{141}	35
5	Arg^{5}	Glu^{139}	98
	Asn^{10}	Cys^{174}	58
	Tyr^{63}	Ser^{142}	51
6	Arg^{5}	Glu^{139}	73
	Arg^{4}	Glu^{139}	30
7	Arg^{5}	Glu^{139}	84
	Arg^{23}	Glu^{139}	42
8	Arg^{5}	Glu^{139}	97
	Arg^{5}	His^{116}	55
	Arg^{4}	His^{116}	36
	Arg^{4}	Glu^{121}	33
9	Arg^{23}	Glu^{139}	99
	Asn^{18}	His^{116}	38
10	Arg^{5}	Glu^{139}	88
	Asn^{10}	His^{116}	52
	Arg^{23}	Glu^{139}	41
	Arg^{23}	Ser^{140}	39

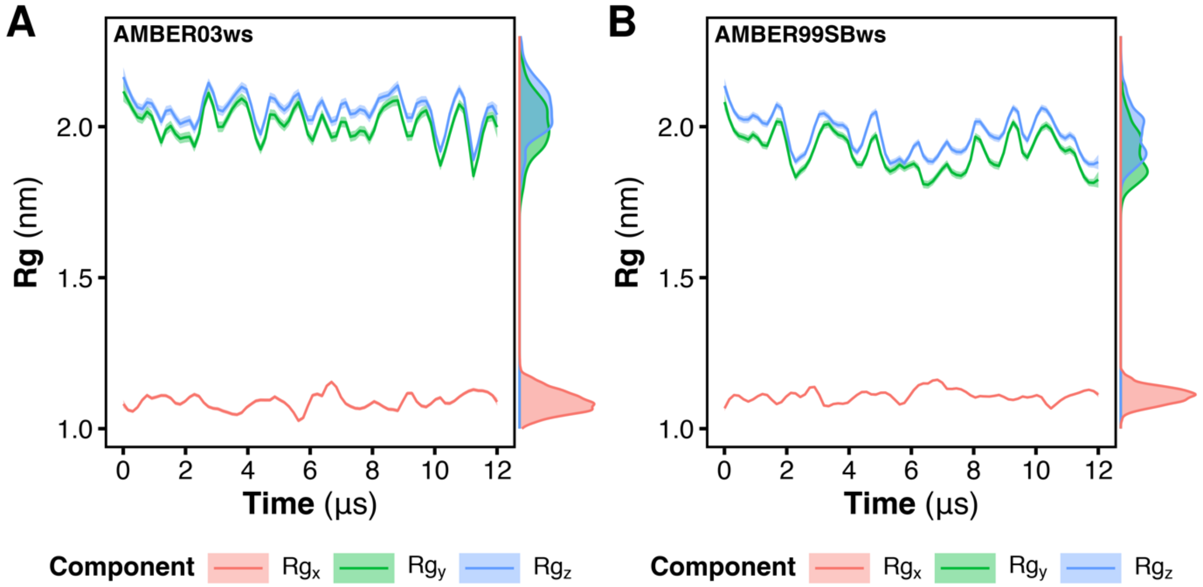

Figure S1. Cumulative trajectories of the radius of gyration components, as obtained from MD simulations performed with the AMBER03ws force field (A) and with the AMBER99SBws force field (B). The distributions of the radius of gyration components are reported as marginal plots.

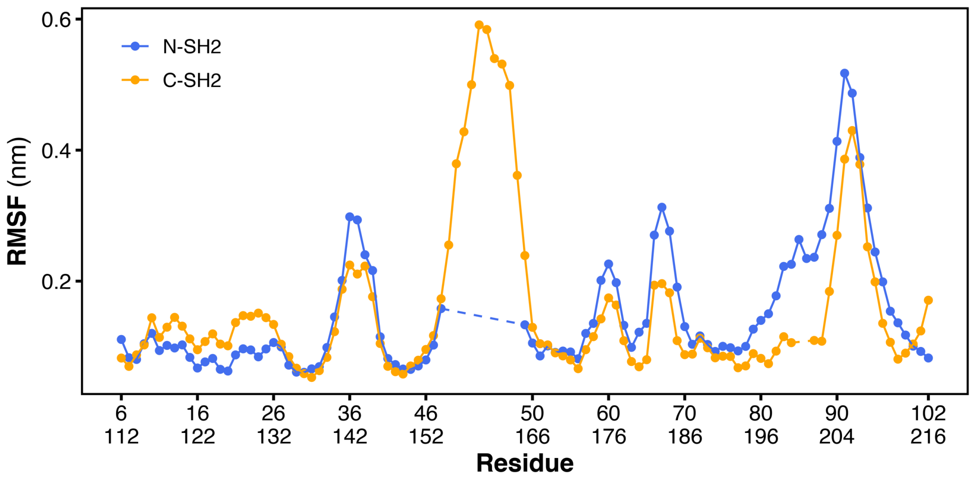

Figure S2. Root mean-squared fluctuations (RMSFs) of the N-SH2 domain (cyan) and the C-SH2 domain (orange) after least-squares fitting over the respective Cα atoms. Amino acid sequences of the domains were aligned after structure superposition of the C-SH2 domain structure over the N-SH2 domain structure.

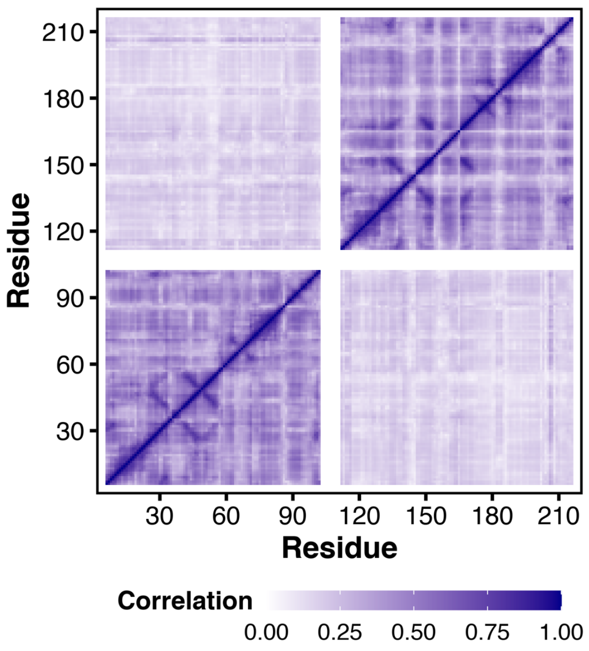

Figure S3. Cross-correlation matrix of the residue-residue displacements from the MD simulations of the tandem SH2 performed with the AMBER99SBws force field. Cross-correlation was calculated from the positions of the Cα atoms after separating the internal local motions of the single domains from the rigid body motions (see Methods).

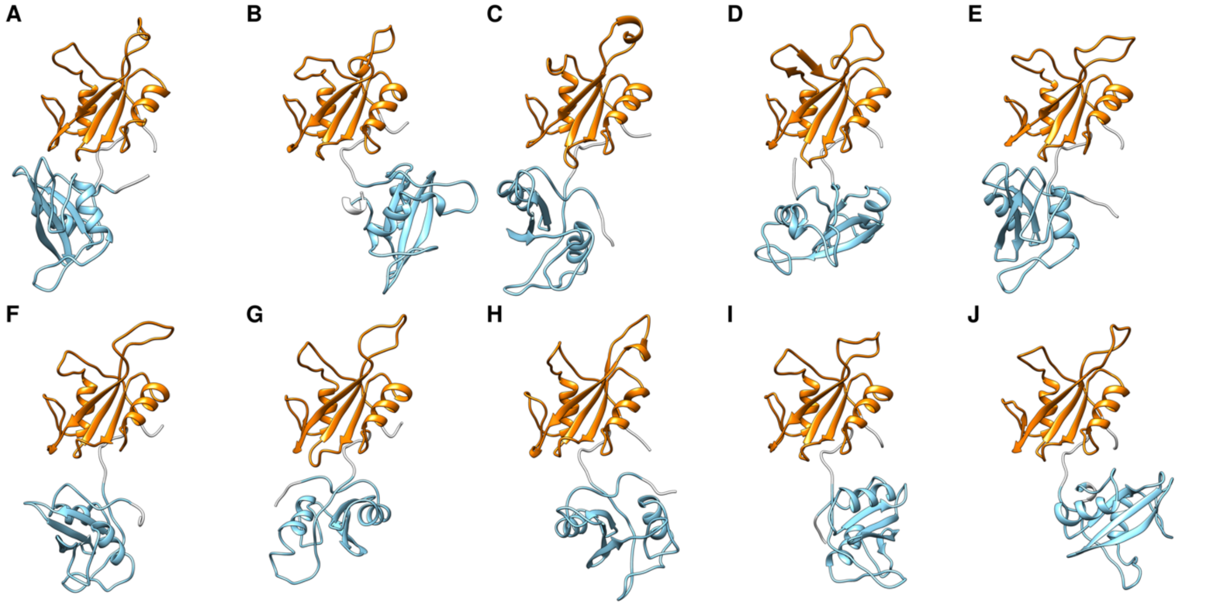

Figure S4. Representative structures of the ten most populated clusters from the MD simulations of the tandem SH2 performed with the AMBER99SBws force field (panels A–J). The N-SH2 and C-SH2 domains are depicted as ribbons and colored in cyan and orange, respectively. The structures were aligned on the C-SH2 domain.

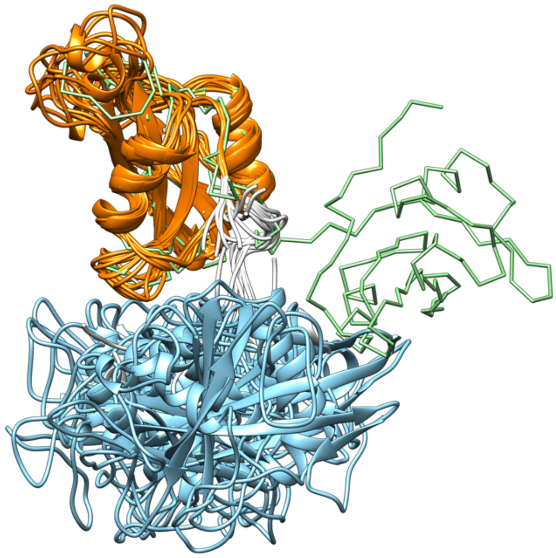

Figure S5. Overlay of the representative structures of the ten most populated clusters from the MD simulations of the tandem SH2 performed with the AMBER99SBws force field. The N-SH2 and C-SH2 domains are depicted as ribbons and colored in cyan and orange, respectively. For comparison, the conformation adopted by the tandem SH2 in autoinhibited SHP2 is represented as a backbone trace colored in light green.

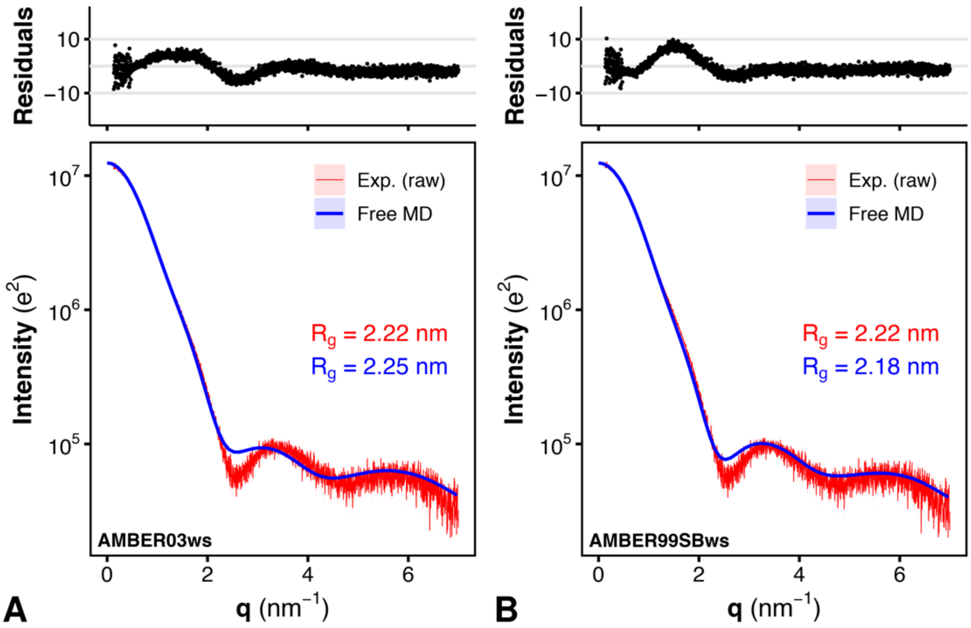

Figure S6. Comparison of the experimental small-angle X-ray scattering (SAXS) curve (red), reported as raw data, with the calculated SAXS curves (blue) of the ensemble of the tandem SH2 structures, obtained from MD simulations performed with the AMBER03ws (A) and the AMBER99SBws force field (B), respectively. Experimental and calculated radii of gyrations (R_g) are reported in each panel. Residuals are plotted as a function of q in the top panels.

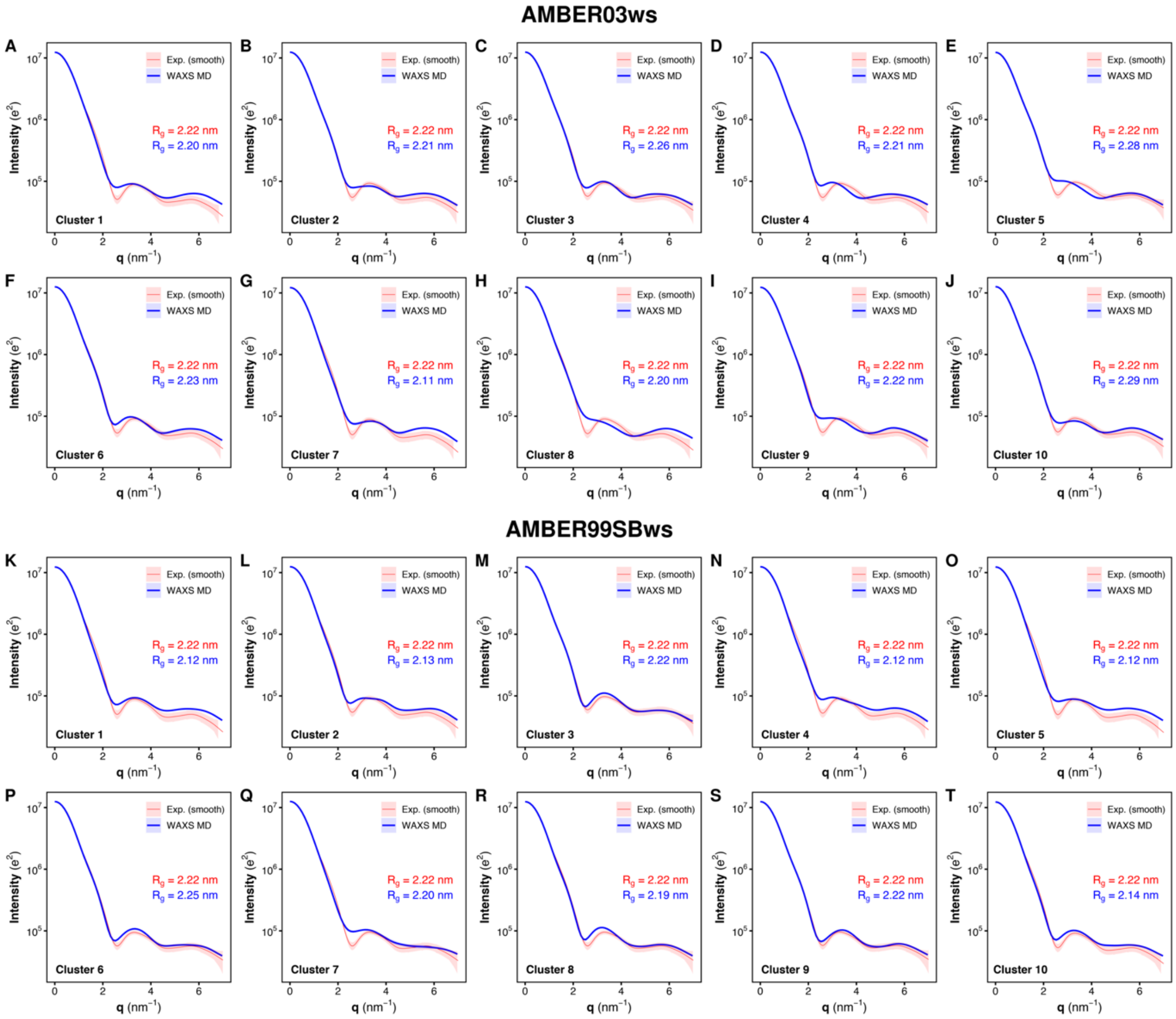

Figure S7. Comparison of the experimental small-angle X-ray scattering (SAXS) curve (red), reported as a smoothed curve, with the SAXS curves calculated from the structure ensembles of each of the ten most populated clusters of the tandem SH2 (blue), obtained from MD simulations performed either with the AMBER03ws force field (panels A–J) or with the AMBER99SBws force field (panels K–T). Experimental and calculated radii of gyrations (R_g) are reported in each panel.

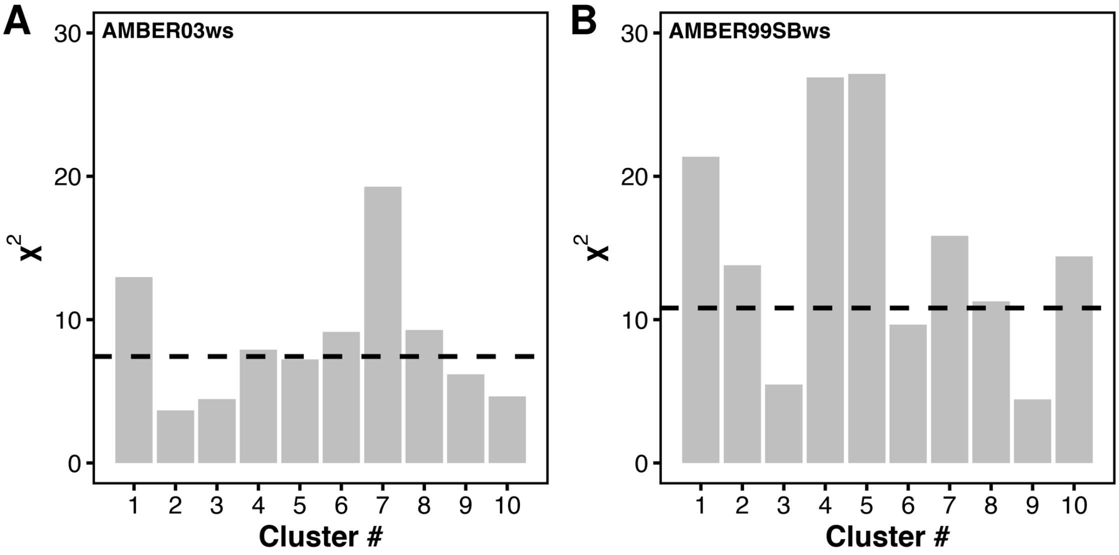

Figure S8. χ^2 values between the experimental small-angle X-ray scattering (SAXS) curve of the tandem SH2 and the calculated SAXS curve from the structure ensembles of each of the ten most populated clusters of tandem SH2, obtained from MD simulations performed either with the AMBER03ws (A) or with the AMBER99SBws force field (B). Dashed lines indicate the corresponding χ^2 value calculated from the entire structural ensembles.

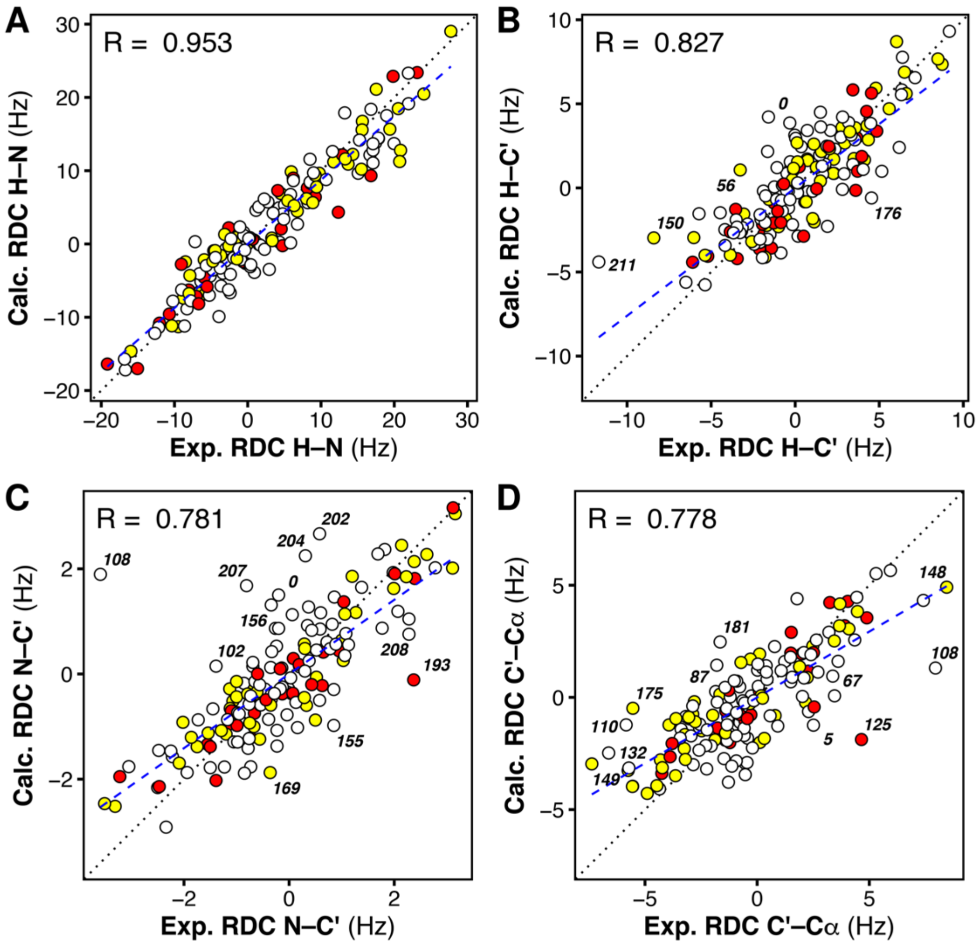

Figure S9. Correlation between the experimental residual dipolar couplings (Exp. RDC) and the back-calculated residual dipolar couplings (Calc. RDC) for each set of measured RDCs (H–N (A), H–C' (B), N–C' (C), C'–Cα (D)), as obtained from the fitting of 20 conformations of tandem SH2 randomly selected from the unrestrained ensemble using the multiple-template/multiple-tensor approach (corresponding to the setup returning the maximum Pearson correlation coefficient in Figure S10). Pearson correlation coefficients *R* for each set of RDCs are shown in each panel. The points with highest deviation are reported with the sequence number of the first residue involved. The points are colored according to the secondary structure of the first residue involved: red (α-helix), yellow (β-sheet), white (coil, other).

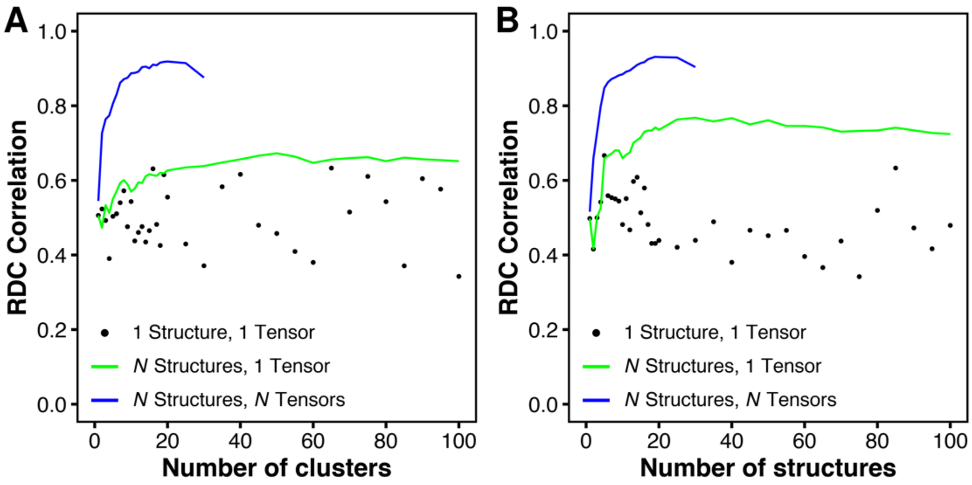

Figure S10. Pearson correlation coefficient between all experimental RDCs and the back-calculated RDCs versus the number of structures in the ensemble. The RDCs were calculated using respectively one structure and one alignment tensor (single-template/single-tensor, black points), one ensemble of structures and one alignment tensor (multiple-template/single-tensor, green line), and one ensemble of structures and one ensemble of alignment tensors (multiple-template/multiple-tensor, blue line). For the single-template/single-tensor approach, one structure was randomly selected from the pool of conformations under evaluation. The calculations were performed (A) on a population-weighted set of central structures from the most populated clusters, and (B) on an unweighted set of structures randomly selected from the ensemble. The analysis demonstrates that the heterogenous ensemble explains the experimental RDCs far better than any single structure.

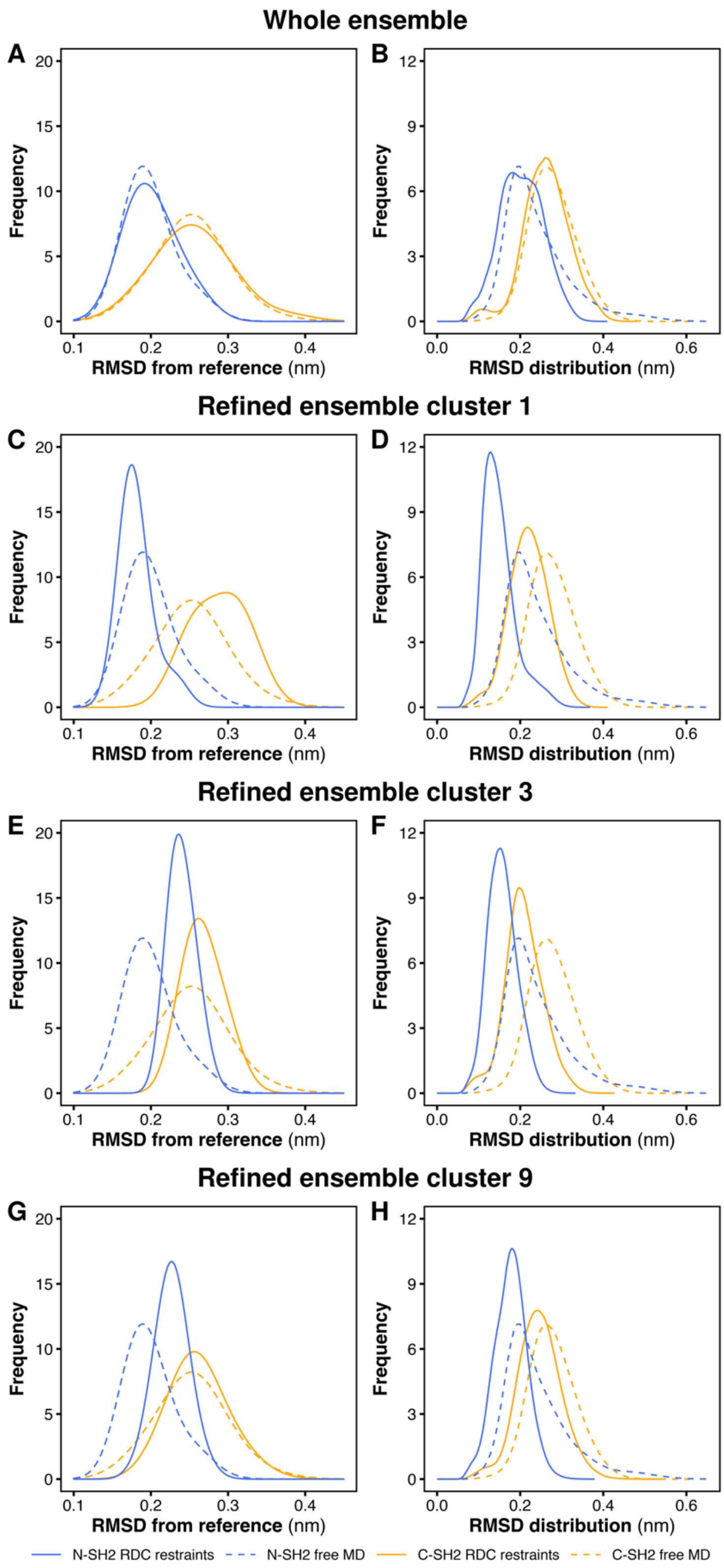

Figure S11. Distribution of the root mean squared deviation (RMSD), from the respective reference individual-domain structures of the N-SH2 domain (blue) and C-SH2 domain (orange) belonging to the tandem SH2 (PDB ID 5df6) in the RDC-restrained (solid lines) or unrestrained (dashed lines) ensembles (panels A, C, E, G). Distribution of the pairwise RMSD across the structures of the N-SH2 domain (blue) and C-SH2 domain (orange) of tandem SH2 in the RDC-restrained (solid lines) or unrestrained (dashed lines) ensembles (panels B, D, F, H). The RDC-restrained ensemble was generated using as starting coordinates 24 randomly selected conformations representing either the entire heterogenous ensemble of MD-derived tandem SH2 structures (panels A, B) or the homogeneous ensembles of cluster 1 (panels C, D), cluster 3 (panels E, F), and cluster 9 (panels G, H). The attempt to fit the RDC data with tandem SH2 structures with a defined relative orientation of the two domains (as in cluster 1, 3 and 9) leads to major perturbations in the conformation of either the N-SH2 (E and G) or the C-SH2 (C) domain.

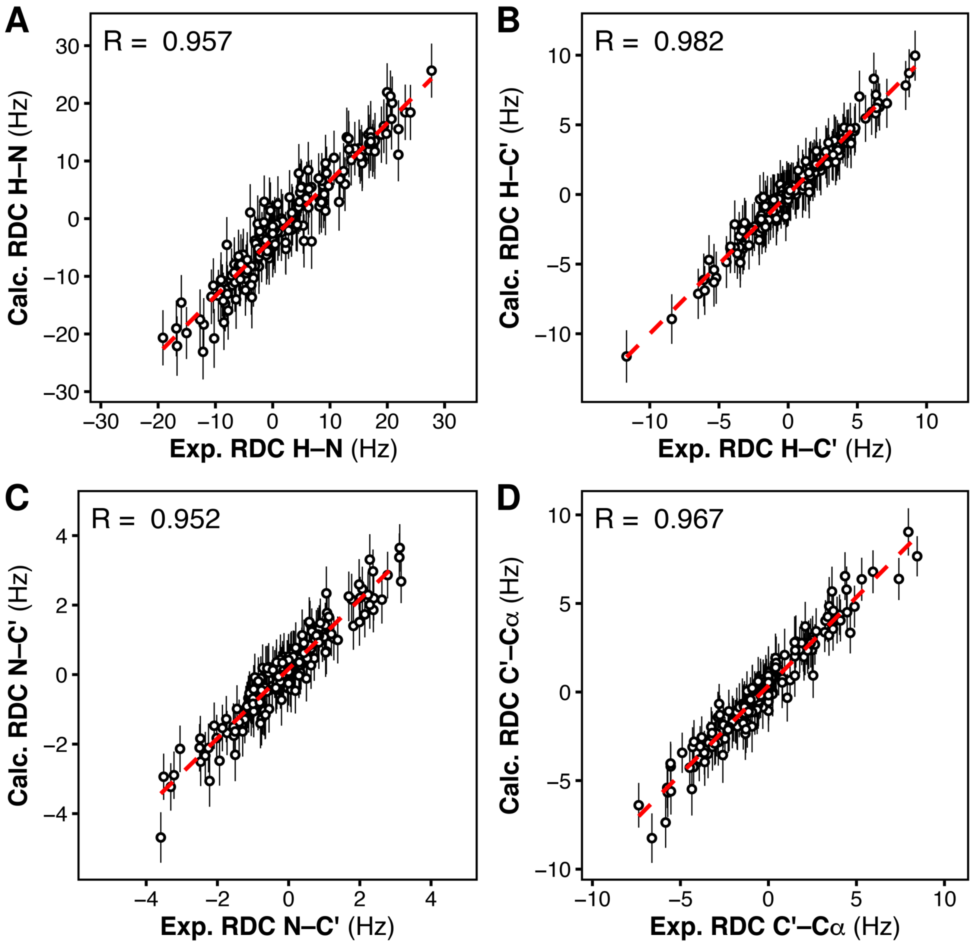

Figure S12. Correlation between the experimental dipolar couplings (Exp. RDC) and the calculated residual dipolar couplings (Calc. RDC), for each type of RDCs, H–N (A), H–C' (B), N–C' (C), C'–Cα (D), as obtained from RDC-restrained multi-replicas MD simulations of the tandem SH2, starting from 24 randomly selected conformations belonging to cluster 1.

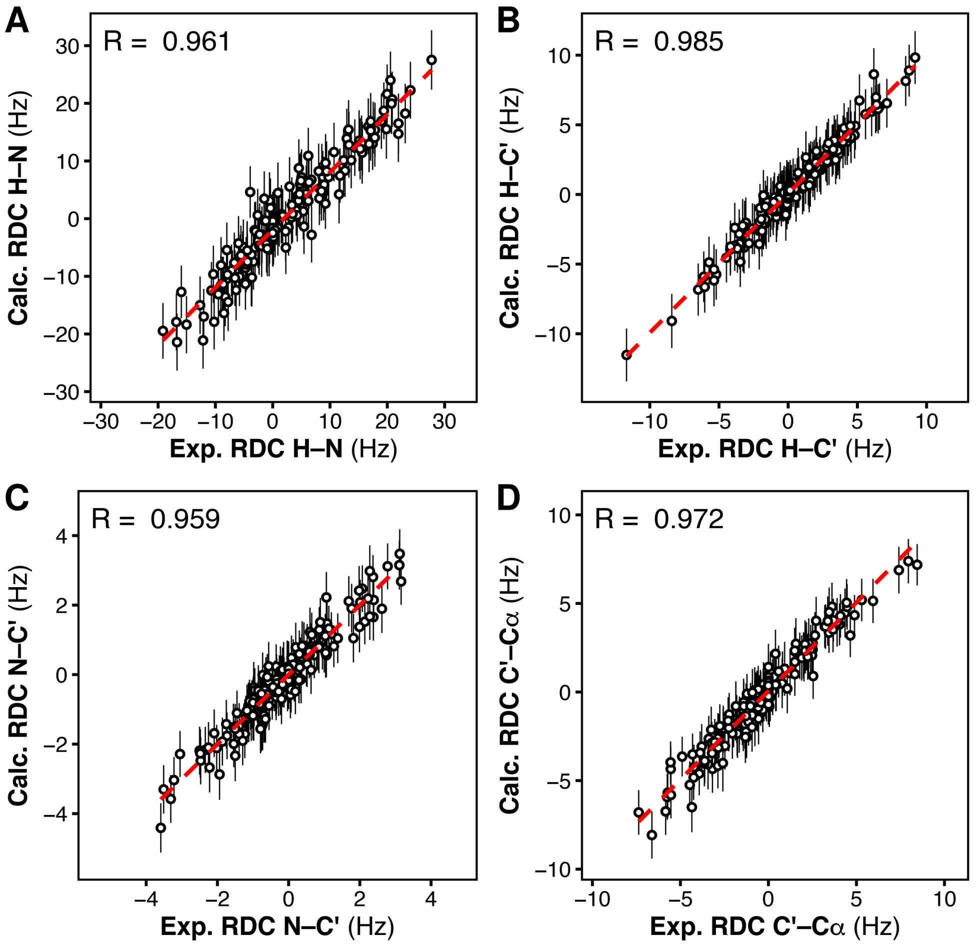

Figure S13. Correlation between the experimental dipolar couplings (Exp. RDC) and the calculated residual dipolar couplings (Calc. RDC), for each type of RDCs, H–N (A), H–C' (B), N–C' (C), C'–Cα (D), as obtained from RDC-restrained multi-replicas MD simulations of the tandem SH2, starting from 24 randomly selected conformations belonging to cluster 3.

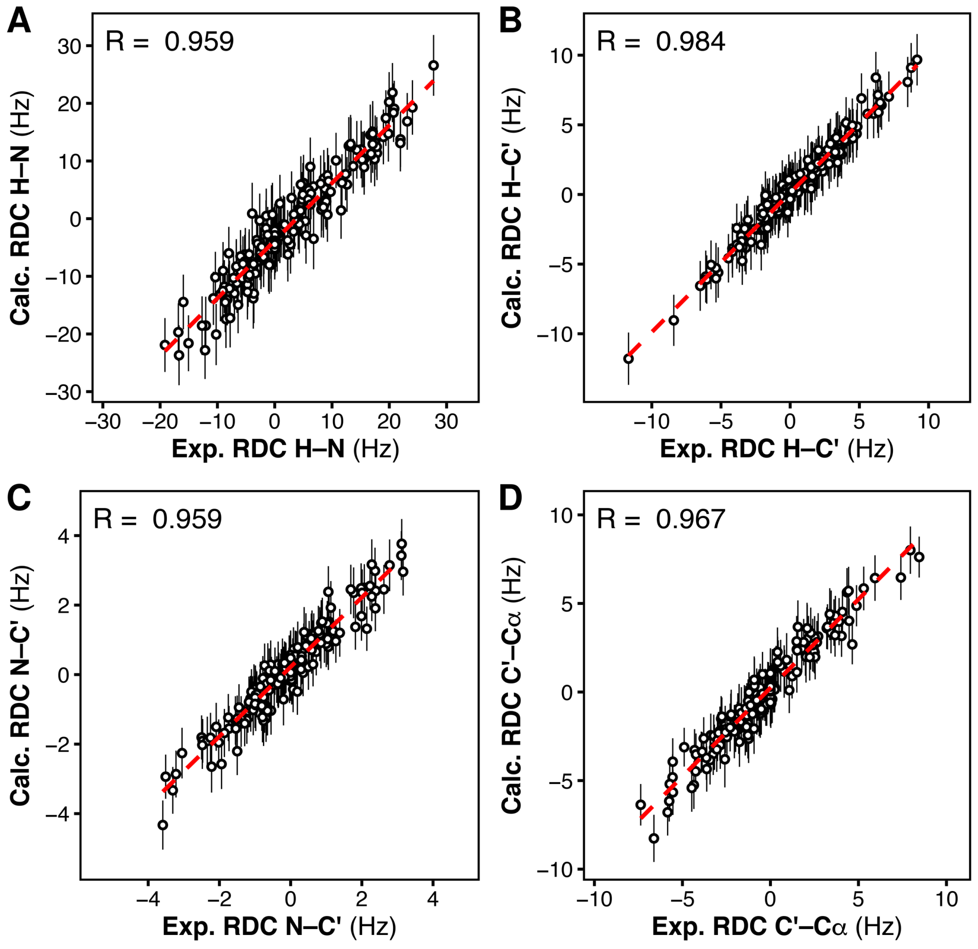

Figure S14. Correlation between the experimental dipolar couplings (Exp. RDC) and the calculated residual dipolar couplings (Calc. RDC), for each type of RDCs, H–N (A), H–C' (B), N–C' (C), C'–Cα (D), as obtained from RDC-restrained multi-replicas MD simulations of the tandem SH2, starting from 24 randomly selected conformations belonging to cluster 9.

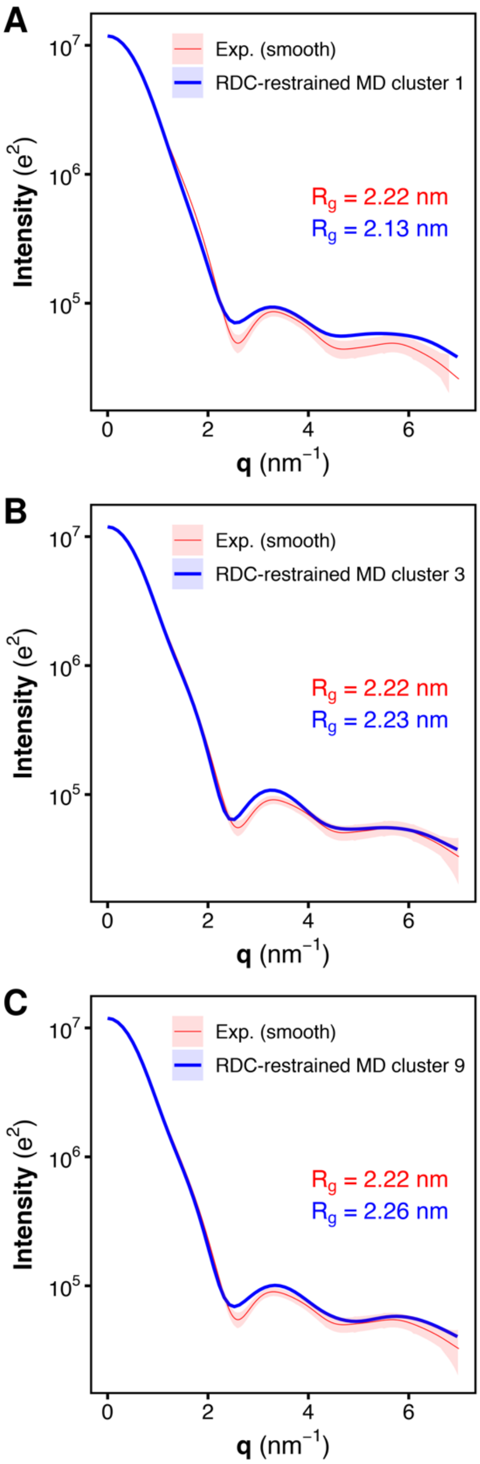

Figure S15. Comparison of the experimental small-angle X-ray scattering (SAXS) curve (red), reported as a smoothed curve, with the calculated SAXS curves (blue) of the RDC-restrained ensemble of tandem SH2, obtained from multi-replica MD simulations starting from 24 conformations representing the ensembles of cluster 1 (A), cluster 3 (B), and cluster 9 (C). Experimental and calculated radii of gyrations (R_g) are reported in each panel.

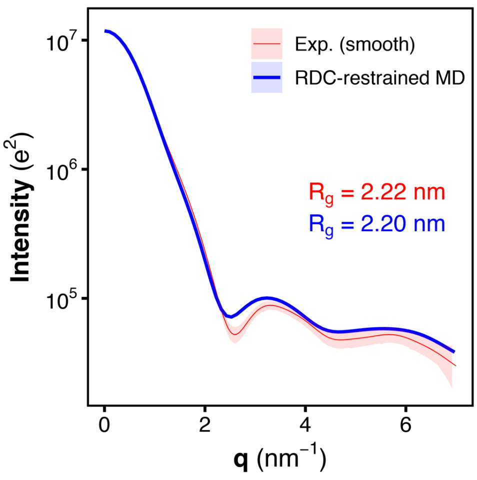

Figure S16. Comparison of the experimental small-angle X-ray scattering (SAXS) curve (red), reported as a smoothed curve, with the calculated SAXS curve (blue) from the RDC-restrained ensemble of the tandem SH2, obtained from multi-replica MD simulations starting from 24 conformations representing the entire MD-derived ensemble. Experimental and calculated radii of gyrations (R_g) are reported.

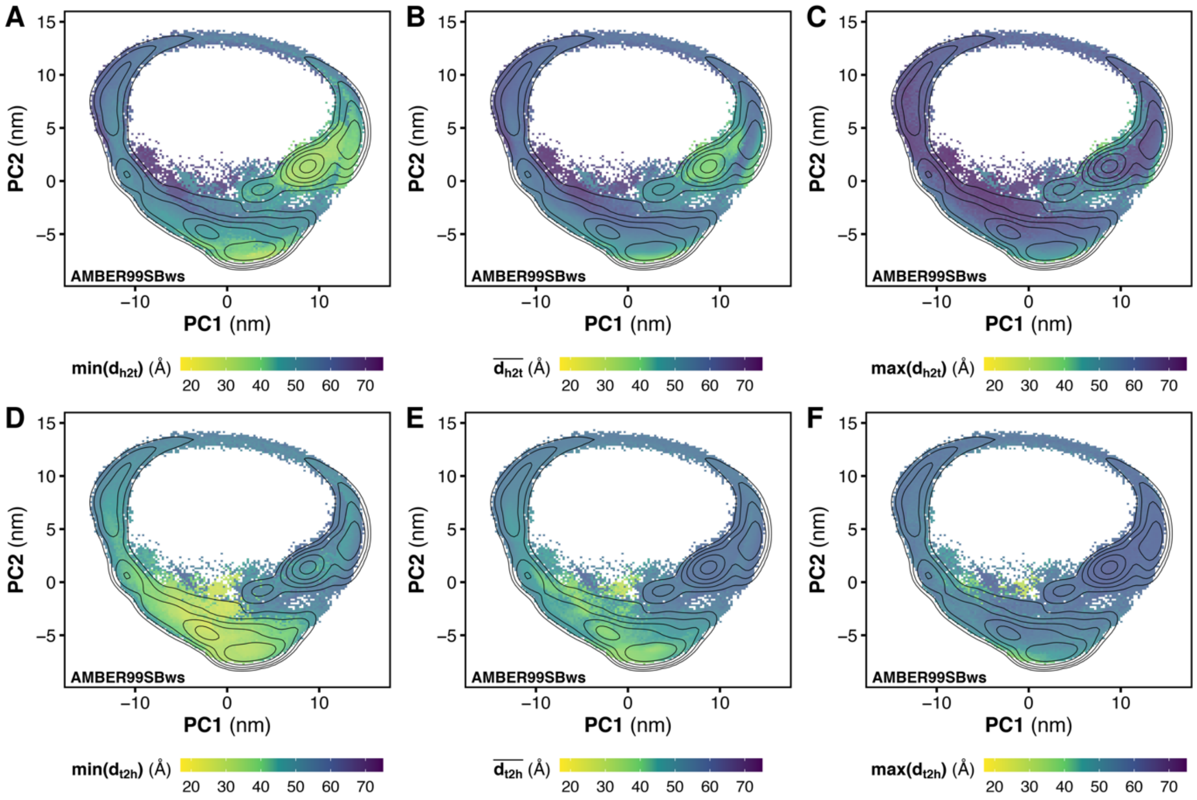

Figure S17. Head-to-tail distance d_{h2t} (panels A–C) and tail-to-head distance d_{t2h} (panels D–F) as a function of the principal components PC1 and PC2, obtained from analysis of MD simulations performed with the AMBER99SBws force field. The free energy landscape is reported as a contour plot (isosurface lines indicating steps of 2 kJ/mol). The smallest (panels A, D), the averaged (panels B, E), and the largest values (panels C, F) of both *h2t* and *t2h* distances are reported as colored maps.

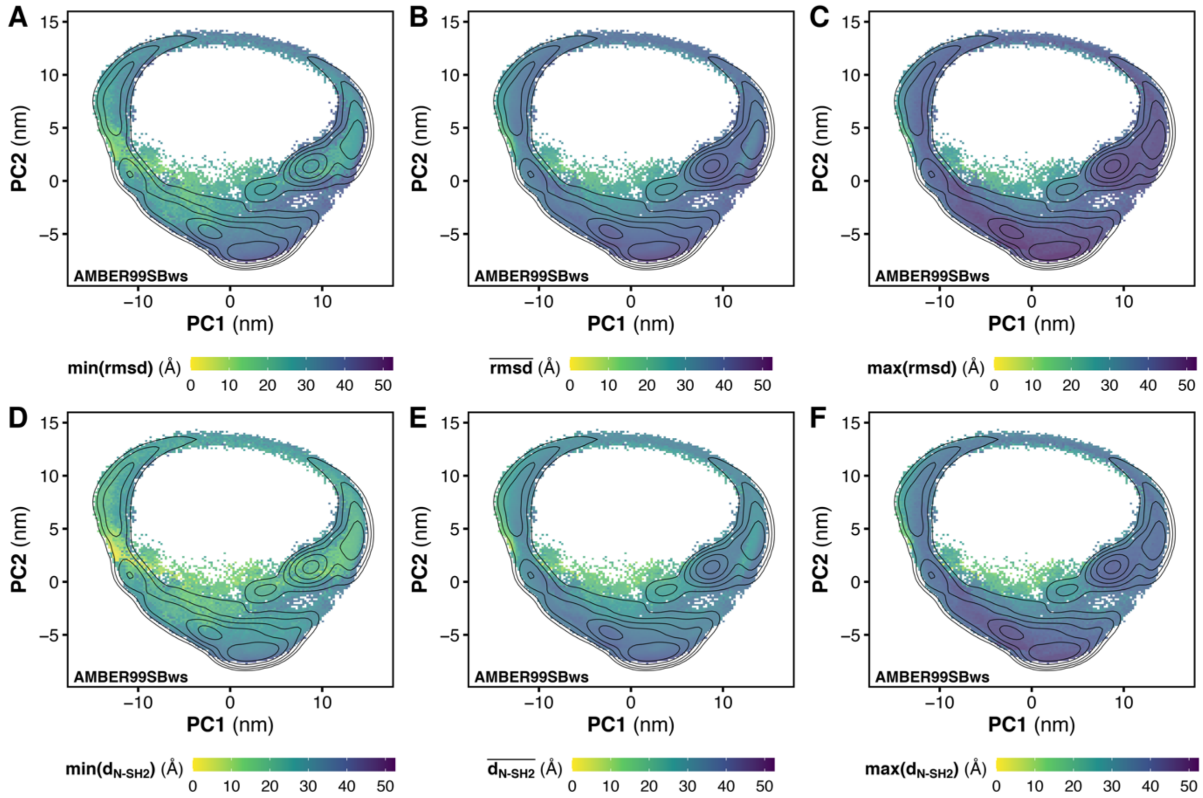

Figure S18. Root mean squared deviation (*rmsd,* panels A–C) and center-of-mass distance ($d_{N\text{-}SH2}$, panels D–F) of the N-SH2 domain relative to the N-SH2 domain in the autoinhibited structure of SHP2, as a function of the principal components PC1 and PC2, as obtained from MD simulations performed with the AMBER99SBws force field. The potential of mean force (PMF) is reported as a contour plot (isosurface lines indicating steps of 2 kJ/mol). The smallest (panels A, D), the averaged (panels B, E), and the largest values (panels C, F) of both *rmsd* and $d_{N\text{-}SH2}$ are reported as colored maps.

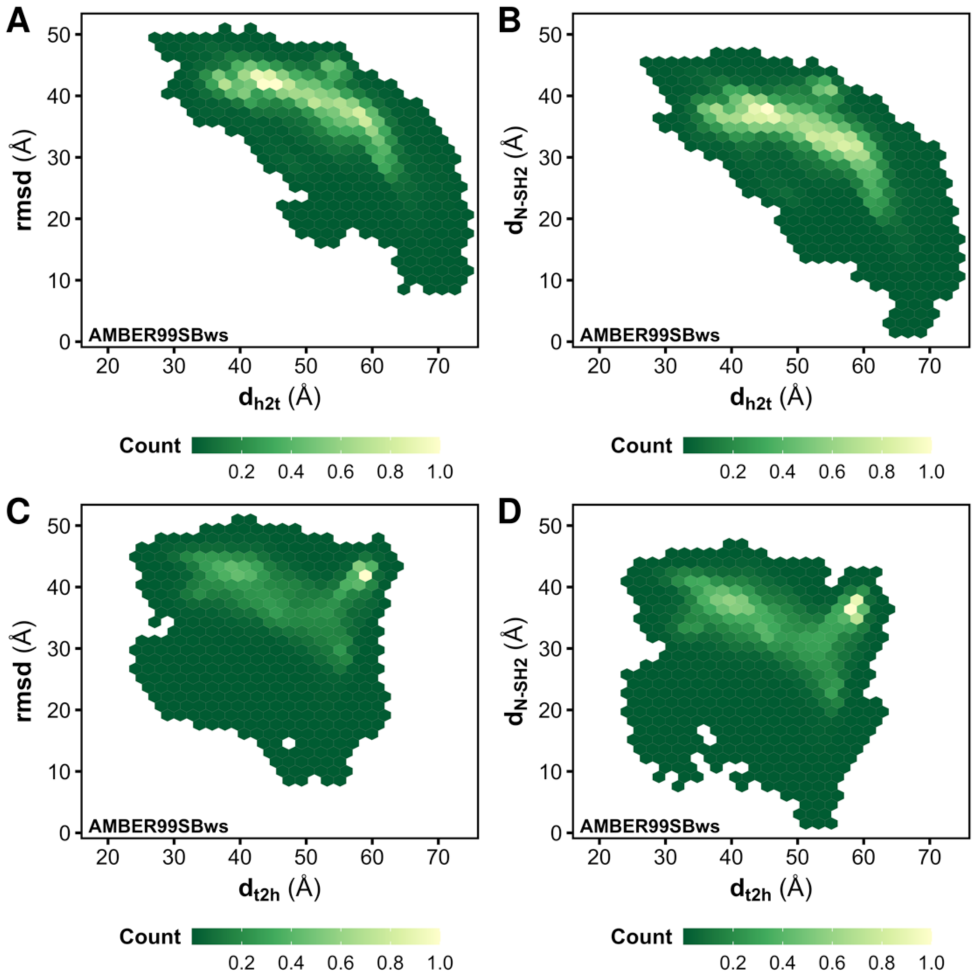

Figure S19. Density maps of the root mean squared deviation (*rmsd,* panel A) and of the center-of-mass distance ($d_{\text{N-SH2}}$, panel B) of the N-SH2 domain relative to the N-SH2 domain in the autoinhibited structure of SHP2, as a function of head-to-tail (d_{h2t}) distance, as obtained from MD simulations performed with the AMBER99SBws force field. Density map of the root mean squared deviation (*rmsd,* panel C) and of the center-of-mass distance ($d_{\text{N-SH2}}$, panel D) of the N-SH2 domain relative to the N-SH2 domain in the autoinhibited structure of SHP2, as a function of tail-to-head (d_{t2h}) distance, as obtained from MD simulations performed with the AMBER99SBws force field.

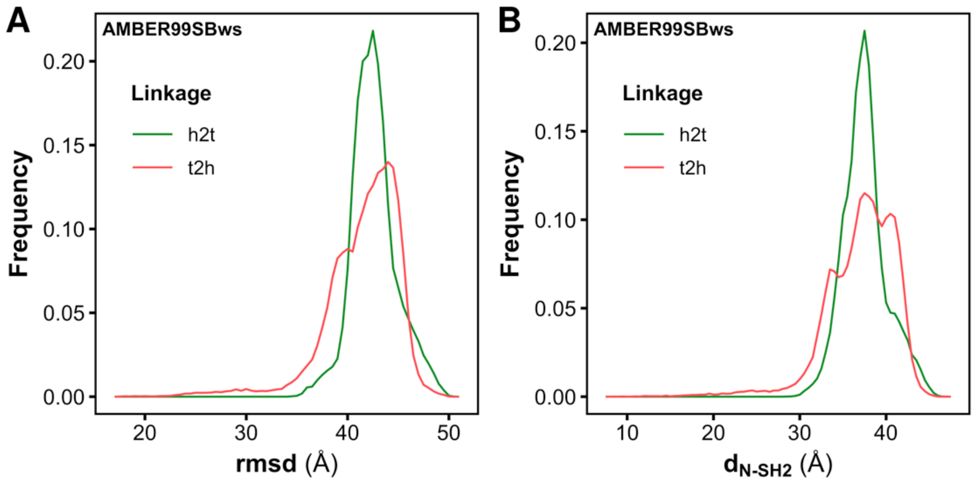

Figure S20. Distributions of the root mean squared deviation (*rmsd*, panel A) and of the center-of-mass distance ($d_{\text{N-SH2}}$, panel B) of the N-SH2 domain relative to the N-SH2 domain in the autoinhibited structure of SHP2, for values of the head-to-tail (*h2t*, green line) and tail-to-head (*t2h*, red line) distances below 40 Å.

METHODS

Sample preparation

The DNA sequence encoding SHP2$^{1\text{-}220}$ (tandem SH2) was cloned into the pETM22 expression vector (European Molecular Biology Laboratory collection), which allows the expression of the recombinant protein as a fusion construct with His_6-tagged thioredoxin for improved solubility. Protein expression was achieved by growing BL21(DE3) *E. coli* at 37 °C in a shaker to an OD_{600} of 0.6-0.8, at which point the culture was rapidly cooled in an ice-water mix and induced with 0.2 mM IPTG at 20 °C. The bacteria were kept growing for 18 hours, after which they were harvested and the cell pellets stored at –20 °C until purification. Preparation of uniformly ^{15}N,^{13}C-labeled protein for NMR studies was achieved by growing the bacteria in M9 minimal medium containing kanamycin (50 μg/ml), $^{15}NH_4Cl$ (1g/liter), and ^{13}C-D-glucose (4g/liter). Isotopes were purchased from Cambridge Isotope Laboratories. For the protein needed in SAXS experiments, the bacteria were grown in Luria-Bertani broth supplemented with kanamycin (50 μg/ml).

On the day of purification, the bacterial pellets were resuspended in wash buffer (1 M NaCl, 50 mM tris (pH 7.6), 2% glycerol, 10 mM imidazole, and 5 mM β-mercaptoethanol) supplemented with one tablet of EDTA-free protease inhibitor cocktail (Roche), 100 μg of lysozyme (Roth), and 50 μg of deoxyribonuclease (NEB). Lysis was performed by sonication, after which the lysate was clarified via centrifugation at 19000 rpm for one hour, and the supernatant was recovered and filtered, before loading it on a HisTrap HP column (GE Healthcare), previously equilibrated with wash buffer. The His_6-tagged thioredoxin-tandem SH2 was eluted with a step gradient of 100% elution buffer (1 M NaCl, 50 mM tris (pH 7.6), 2% glycerol, 500 mM imidazole, and 5 mM β-mercaptoethanol). Cleavage of the thioredoxin tag was performed overnight at 4 °C with 3C protease, while excess imidazole was removed by dialysis against 2 liters of wash buffer. Purification proceeded with a second HisTrap step

followed by size-exclusion chromatography on a HiLoad 16/600 Superdex 200pg column (GE Healthcare), previously equilibrated with NMR/SAXS buffer (100 mM MES (pH 6.8), 150 mM NaCl, 5 mM DTT, 0.01% w/v sodium azide). Finally, the protein was concentrated to the desired value and either used directly or flash-frozen with liquid nitrogen for long-term storage at –80 °C.

NMR spectroscopy

NMR experiments for extraction of RDCs were collected on uniformly ^{13}C,^{15}N-labeled protein samples at a concentration of ~500 µM, dissolved in NMR buffer [100 mM MES (pH 6.8), 150 mM NaCl, 5 mM DTT, 0.01% w/v sodium azide, 10% v/v D2O], and loaded into 5-mm NMR tubes (sample volume ~550 µL). Spectra were measured at a temperature of 298 K on an 850-MHz Bruker AVIII-HD spectrometer equipped with an inverse HCN CP-TCI cryogenic probe-head and running Bruker Topspin software (v3.2).

Each type of NMR experiment was recorded on isotropic and anisotropic (aligned) samples. The anisotropic sample was prepared by addition of Pf1 filamentous bacteriophage (ASLA Biotech, Latvia) to a final concentration of ~13 mg/ml. The formation and homogeneity of the anisotropic phase were confirmed by inspection of the D2O ^{2}H spectrum, which showed a well-resolved doublet with a splitting of 8.3 Hz.

Each type of RDC (H–N, N–C', H–C' and C'–Cα) was calculated as difference between the respective isotropic and anisotropic doublet-splittings. All doublet-splittings were extracted from IPAP-type (in-phase/anti-phase) spectra; in this approach, each RDC experiment comprises two sub-spectra ("in-phase" and "anti-phase"), in which the relevant doublet appears as either in-phase (the two doublet peaks have the same sign) or anti-phase (the two doublet peaks have opposite sign). Two new sub-spectra ("upfield" and "downfield") that contain either one or other of the two doublet peaks were generated by taking the sum and difference of the in-phase and anti-phase sub-spectra. The peak-positions for calculation of the doublet-

splittings were then measured from the upfield and downfield sub-spectra. H–N splittings were extracted from 2D IPAP–^{15}N-HSQC spectra,[1] and recorded with Hα/Hβ band-selective decoupling for ^{15}N chemical-shift evolution.[2] C'–Cα splittings were recorded from 3D IPAP–HNCO[J-CA] spectra.[3] N–C' and H–C' splittings were both extracted from 2D IPAP-E.COSY–^{15}N-HSQC spectra. In this experiment, the in-phase/anti-phase sub-spectra were generated by either refocusing or evolving ^{15}N transverse magnetization with respect to the N–C' coupling prior to the indirect evolution period. The doublet components in the resultant 2D spectrum were separated by the N–C' splitting in the indirect dimension and by the H–C' splitting in the acquisition (direct) dimension. All spectra were processed with NMRPipe (v10.1).[4] Peak-positions were determined with CcpNmr Analysis.[5]

SAXS experiments

Small-angle X-ray scattering (SAXS) data were collected at the P12 beamline at the Petra III storage ring at DESY (Deutsches Elektronen-Synchrotron) in Hamburg (Germany). Six different tandem SH2 samples were prepared at six different concentrations in SAXS buffer [100 mM MES (pH 6.8), 150 mM NaCl, 5 mM DTT, 0.01% w/v sodium azide] supplemented with additional 10 mM DTT immediately before the measurements to minimize radiation damage. Data collection was carried out at 20 °C with exposure of 10 frames each of 1 s duration. Curve analysis and extrapolation at infinite dilution were done by PRIMUS in ATSAS.[6]

MD simulations of the tandem SH2 in solution

Molecular dynamics simulations were performed for the tandem SH2 domains of wild-type SHP2 in apo form (SHP2$^{1\text{-}220}$, corresponding to sequence ranges from Met^{1} to Arg^{220}), except for the N-terminus that was modified by adding two residues, Gly^{-1} and Pro^{0}, and replacing Thr^{2} with alanine. The initial atomic coordinates were derived from the

crystallographic structure of the *PTPN11* tandem SH2 domains in complex with TXNIP peptides (PDB ID 5df6).[7] Missing or incomplete residues (strands Gly^{-1}–Ser^{3}, Asn^{161}–Gly^{163}, Glu^{176}–Leu^{177}) were modeled by Molecular Operative Environment (MOE).[8] Tandem SH2 domains were put at the center of a dodecahedron box, large enough to contain the protein and at least 2.1 nm of solvent on all sides. The system was solvated with ~39500 explicit TIP4P/2005 water molecules,[9] and one Na^+ ion was added to neutralize the simulation box. All MD simulations were performed with the GROMACS software package,[10] using the AMBER03ws or AMBER99SBws force fields.[11] Long range electrostatic interactions were calculated with the particle-mesh Ewald (PME) approach.[12] A cutoff of 1.2 nm was applied to the direct-space Coulomb and Lennard-Jones interactions. Bond lengths and angles of water molecules were constrained with the SETTLE algorithm,[13] and all other bonds were constrained with LINCS.[14] The solvent was relaxed by energy minimization followed by 100 ps of MD at 298 K, while restraining protein coordinates with a harmonic potential. Then the system was minimized without restraints. Starting from the last system structure, 12 simulations were spawned, after generating the initial velocities at 50 K from different seeds according to the Maxwell distribution. The temperature was raised to 298 K in 10 ns, in a stepwise manner, while the pressure was set to 1 bar using the weak-coupling barostat.[15] Finally, 12 independent production simulations of 1.05 μs were performed. The pressure was set to 1 bar using the Parrinello-Rahman barostat.[16] The temperature was controlled at 298 K using velocity rescaling with a stochastic term.[17] The first 50 ns of each simulation were discarded, and the analyses were performed on a cumulative trajectory of 12 μs.

First-order water-mediated hydrogen bonds were analyzed by MDAnalysis tools.[18] The cross-correlation between the N-SH2 and C-SH2 domains was calculated by Linear Mutual Information (LMI) method[19] from the positions of the Cα atoms after separating the internal local motions of the single domains from the rigid body motions.[20]

SAXS calculations from explicit solvent MD simulations

SAXS curves were computed from the MD simulations using explicit-solvent SAXS calculations.[21] Accordingly, all explicit water molecules and ions within a predefined distance from the protein contributed to the SAXS calculations, as defined by a spatial envelope.[21] A distance of the envelope from the solute atoms (7 Å) was chosen to ensure bulk-like water at the envelope surface.[21] A density correction was applied to fix the bulk water density to the experimental value of 334 e nm^{-3}.[21] The buffer-subtracted SAXS curve was computed from the scattering of atoms inside the envelope volume, as taken from MD simulation frames of two systems: *i)* containing the tandem SH2 in solvent and *ii)* containing pure solvent.[21]

Back-calculation of RDC from tandem SH2 structures

RDCs were calculated after an alignment tensor best-fitting with the experimental RDCs using singular value decomposition (SVD).[22] Calculations were performed by calcTensor (for the single-template/single-tensor and the multiple-template/single-tensor approach) and calcETensor (for the multiple-template/multiple-tensor approach) helper programs of Xplor-NIH suite[23] on a maximum of 100 conformations of tandem SH2, extracted from the AMBER99SBws simulations in solution.

RDC-restrained MD simulations

Langevin MD simulations were performed using the AMBER99SBws force field,[11] with the TIP4P/2005 water model,[9] which had previously provided a structure ensemble in agreement with experimental SAXS curve. The system and the simulation setup were the same as the previous simulations of the tandem SH2 in solution, with the exception of the integrator and the temperature bath. RDCs were applied as ensemble averaged structural restraints using the tensor-free Θ method.[24] The ensemble was composed by 24 parallel replicas, whose initial structures and velocities were randomly taken from the restraint-free ensemble. We selected a

set of experimental RDCs for the H–N, N–C', H–C' and C'–Cα bond vectors of all residues (in total, we used 864 restraints). Plumed 2.7 libraries were used to introduce linear RDC restraints (force constant k = 2000 kJ mol^{-1} nm^{-1}).[25] Starting from a restraint-free simulation of 2 ns, the system was equilibrated introducing the restraints in a step-wise manner (force constant was increased of 20 kJ mol^{-1} nm^{-1} every 1 ns), for a total equilibration time of 102 ns. Finally, a productive run of 100 ns was performed.

REFERENCES

1. M. Ottiger, F. Delaglio and A. Bax, Measurement ofJand Dipolar Couplings from Simplified Two-Dimensional NMR Spectra, *J. Magn. Reson.*, 1998, **131**, 373-378.
2. L. Yao, J. Ying and A. Bax, Improved accuracy of 15N–1H scalar and residual dipolar couplings from gradient-enhanced IPAP-HSQC experiments on protonated proteins, *J. Biomol. NMR*, 2009, **43**, 161-170.
3. P. Permi, P. R. Rosevear and A. Annila, A set of HNCO-based experiments for measurement of residual dipolar couplings in 15N, 13C, (2H)-labeled proteins, *J. Biomol. NMR*, 2000, **17**, 43-54.
4. F. Delaglio, S. Grzesiek, G. W. Vuister, G. Zhu, J. Pfeifer and A. Bax, NMRPipe: A multidimensional spectral processing system based on UNIX pipes, *J. Biomol. NMR*, 1995, **6**, 277-293.
5. W. F. Vranken, W. Boucher, T. J. Stevens, R. H. Fogh, A. Pajon, M. Llinas, E. L. Ulrich, J. L. Markley, J. Ionides and E. D. Laue, The CCPN data model for NMR spectroscopy: Development of a software pipeline, *Proteins*, 2005, **59**, 687-696.
6. M. V. Petoukhov, D. Franke, A. V. Shkumatov, G. Tria, A. G. Kikhney, M. Gajda, C. Gorba, H. D. T. Mertens, P. V. Konarev and D. I. Svergun, New developments in the

ATSAS program package for small-angle scattering data analysis, *J. Appl. Crystallogr.*, 2012, **45**, 342-350.

7. Y. Liu, J. Lau, W. Li, W. Tempel, L. Li, A. Dong, A. Narula, S. Qin and J. Min, Structural basis for the regulatory role of the PPxY motifs in the thioredoxin-interacting protein TXNIP, *Biochem. J.*, 2016, **473**, 179-187.
8. Molecular Operating Environment (MOE) software, Chemical Computing Group Inc., 2014.
9. J. L. F. Abascal and C. Vega, A general purpose model for the condensed phases of water: TIP4P/2005, *J. Chem. Phys.*, 2005, **123**, 234505.
10. D. Van Der Spoel, E. Lindahl, B. Hess, G. Groenhof, A. E. Mark and H. J. Berendsen, GROMACS: fast, flexible, and free, *J. Comput. Chem.*, 2005, **26**, 1701-1718.
11. R. B. Best, W. Zheng and J. Mittal, Balanced Protein–Water Interactions Improve Properties of Disordered Proteins and Non-Specific Protein Association, *J. Chem. Theory Comput.*, 2014, **10**, 5113-5124.
12. T. Darden, D. York and L. Pedersen, Particle Mesh Ewald: an Nlog(N) method for Ewald sum in large systems, *J. Chem. Phys.*, 1993, **98**, 10089-10092.
13. S. Miyamoto and P. A. Kollman, SETTLE: An analytical version of the SHAKE and RATTLE algorithms for rigid water models, *J. Comput. Chem.*, 1992, **13**, 952–962.
14. B. Hess, H. Bekker, H. J. C. Berendsen and J. G. E. M. Fraaije, LINCS: a linear constraint solver for molecular simulations, *J. Comput. Chem.*, 1997, **18**, 1463-1472.
15. H. J. C. Berendsen, J. P. M. Postma, W. F. Van Gunsteren, A. Di Nola and J. R. Haak, Molecular dynamics with coupling to an external bath, *J. Chem. Phys.*, 1984, **81**, 3684-3690.
16. M. Parrinello and A. Rahman, Polymorphic transitions in single crystals: A new molecular dynamics method, *J. Appl. Phys.*, 1981, **52**, 7182–7190.

17. G. Bussi, D. Donadio and M. Parrinello, Canonical sampling through velocity rescaling, *J. Chem. Phys.*, 2007, **126**, 14101.

18. N. Michaud-Agrawal, E. J. Denning, T. B. Woolf and O. Beckstein, MDAnalysis: A toolkit for the analysis of molecular dynamics simulations, *J. Comput. Chem.*, 2011, **32**, 2319-2327.

19. O. F. Lange and H. Grubmüller, Generalized correlation for biomolecular dynamics, *Proteins*, 2006, **62**, 1053-1061.

20. A. Roy, D. P. Hua and C. B. Post, Analysis of Multidomain Protein Dynamics, *J. Chem. Theory Comput.*, 2016, **12**, 274-280.

21. P.-c. Chen and Jochen S. Hub, Validating Solution Ensembles from Molecular Dynamics Simulation by Wide-Angle X-ray Scattering Data, *Biophys. J.*, 2014, **107**, 435-447.

22. J. A. Losonczi, M. Andrec, M. W. F. Fischer and J. H. Prestegard, Order Matrix Analysis of Residual Dipolar Couplings Using Singular Value Decomposition, *J. Magn. Reson.*, 1999, **138**, 334-342.

23. C. D. Schwieters, J. J. Kuszewski, N. Tjandra and G. Marius Clore, The Xplor-NIH NMR molecular structure determination package, *J. Magn. Reson.*, 2003, **160**, 65-73.

24. C. Camilloni and M. Vendruscolo, A Tensor-Free Method for the Structural and Dynamical Refinement of Proteins using Residual Dipolar Couplings, *J. Phys. Chem. B*, 2015, **119**, 653-661.

25. G. A. Tribello, M. Bonomi, D. Branduardi, C. Camilloni and G. Bussi, PLUMED 2: New feathers for an old bird, *Comput. Phys. Commun.*, 2014, **185**, 604-613.
